# Chia seeds oil ameliorate chronic immobilization stress-induced neurodisturbance in rat brains via activation of the antioxidant/anti-inflammatory/antiapoptotic signaling pathways

**DOI:** 10.1038/s41598-023-49061-w

**Published:** 2023-12-16

**Authors:** Norhan E. Khalifa, Ahmed E. Noreldin, Asmaa F. Khafaga, Mohamed El-Beskawy, Eman Khalifa, Ali H. El-Far, Abdel-Hasseb A. Fayed, Abdeldayem Zakaria

**Affiliations:** 1 Department of Physiology, Faculty of Veterinary Medicine, Matrouh University, Matrouh, 51511 Egypt; 2 Department of Histology and Cytology, Faculty of Veterinary Medicine, Damanhour University, Damanhour, 22511 Egypt; 3 Department of Pathology, Faculty of Veterinary Medicine, Alexandria University, Edfina, 22758 Egypt; 4 Department of Animal Medicine, Faculty of Veterinary Medicine, Matrouh University, Matrouh, 51511 Egypt; 5 Department of Microbiology, Faculty of Veterinary Medicine, Matrouh University, Matrouh, 51511 Egypt; 6 Department of Biochemistry, Faculty of Veterinary Medicine, Damanhour University, Damanhour, 22511 Egypt; 7 Department of Physiology, Faculty of Veterinary Medicine, Alexandria University, Edfina, 22758 Egypt

**Keywords:** Biochemistry, Neuroscience, Physiology, Neurology

## Abstract

Chronic immobilization stress plays a key role in several neuropsychiatric disorders. This investigation assessed the possible ameliorative effect of chia seed oil (CSO) against the neurodisturbance-induced in rats by chronic immobilization. Rats were randomly allocated into control, CSO (1 ml/kg b.wt./orally), restrained (6 h/day), CSO pre-restraint, and CSO post-restraint for 60 days. Results revealed a significant reduction in serum corticosterone level, gene expression of corticotrophin-releasing factor, pro-inflammatory cytokines, and oxidative biomarkers in restrained rats treated with CSO. The histopathological findings revealed restoring necrosis and neuronal loss in CSO-treated-restraint rats. The immunohistochemical evaluation revealed a significant reduction in the immuno-expression of caspase-3, nuclear factor kappa B, interleukin-6, and cyclooxygenase-2 (COX-2), and an elevation of calbindin-28k and synaptophysin expression compared to non-treated restraint rats. The molecular docking showed the CSO high affinity for several target proteins, including caspase-3, COX-2, corticotropin-releasing hormone binding protein, corticotropin-releasing factor receptors 1 and 2, interleukin-1 receptor types 1 and 2, interleukin-6 receptor subunits alpha and beta. In conclusion, CSO emerges as a promising candidate against stress-induced brain disruptions by suppressing inflammatory/oxidative/apoptotic signaling pathways due to its numerous antioxidant and anti-inflammatory components, mainly α-linolenic acid. Future studies are necessary to evaluate the CSO therapeutic impacts in human neurodisturbances.

## Introduction

There is plenty of cumulative scientific evidence connecting stress to poor health consequences that disturb individuals' life quality^1^. Stress is a non-specific neurohormonal and psychobiological phenomenon exhibited when facing a hazard to boost survival chances. While mild stress has an adaptive function and is essential for individual existence, prolonged or intense stress induces injury to brain tissues^2,3^. Recently, a diversity of environmental and stressful stimuli has been described to change behavior patterns, alter neurotransmitter production, and induce oxidative injury in distinct brain regions^4,5^.

Under stress conditions, the stress response has developed to maintain homeostasis^6^. The neuroendocrine signaling system that is activated as a stress response includes two key integral components, the sympathetic adrenomedullary system (SAS) and the hypothalamic-pituitary-adrenocortical (HPA) axis^7^. The HPA axis's neuroendocrine response is mirrored by glucocorticoids (GCs) released by the adrenal cortex. These GCs influence various physiological functions, mobilizing energy stores, causing proteolysis and lipolysis, enhancing vasoconstriction by the autonomic nervous system, inhibiting reproductive function, and modifying stress-related behaviors to permit homeostasis^8^. Among body organs, the brain tissues are highly susceptible to GCs (cortisol and corticosterone in humans and rodents, respectively) and other stress-associated endocrine secretions that are critical for mental health and cognition^9^. Furthermore, chronic stimulation of the HPA axis causes adrenal hyperplasia and hypertrophy and consequent upsurges glucocorticoid, which initiates apoptosis of the hippocampus neurons^10^.

The main objective of contemporary medicine is to recognize elements that progress neurodegeneration in the brain. There are numerous theories concerning the mechanisms that initiate brain cell damage and death in neurodegenerative disorders, such as alteration in energy metabolism, excitotoxicity induced by excitatory amino acids, and oxidative stress^11^. The neural tissue is highly susceptible to reactive oxygen species (ROS)-induced-oxidative damage because of its large proportion of oxidized fatty acids content, high rate of oxygen utilization, and limited antioxidant enzyme levels^12^.

Immobilization stress triggers long-term structural and functional alterations in several brain areas^13^. The lack of mechanical function (disuse) caused by immobilization or restraint stress leads to ROS overproduction and overaccumulation, causing oxidative stress status, which is a disequilibrium between the free radical species generation and scavenging, for instance, superoxide ion and peroxide radical^14^. Moreover, the elevated metabolic rate creates excess free radicals, impairing the antioxidant enzyme system and ROS creation^15^, causing oxidative damage to various cellular molecules, for example, lipids, nucleic acids, and proteins^16^.

Now, global attention is directed toward herbal medication to cure several diseases and disorders owing to its bioactive compound content^5^. Numerous natural antioxidants display beneficial progress against stress-induced cognitive complications^17^. Chia (*Salvia hispanica*) is known as a herbaceous plant produced annually. Chia seeds have been eaten since ancient times owing to their nutritional properties and therapeutical aspects^18^. The seeds contain about 18–24% high-quality protein, 25–40% oil, and 30–34% dietary fiber^19^. It is worthwhile stating that α-linolenic acid (ALA) in the chia seeds represents about 60% of total fat content, which is significantly greater than in flaxseeds^20^. Because chia seeds are gluten-free, it was proven that they would not induce any allergies^21^. Also, minerals (phosphorus, potassium, calcium, among many others) and vitamins (mostly B complex) have been mentioned appreciably. Concerning other bioactive compounds, it is also a great source of natural antioxidants, including kaempferol, quercetin, caffeic, and chlorogenic acids^19,22^. Several studies revealed that whole and ground chia seeds and mucilage have potent antioxidant activity, which enhances the antioxidant status and attenuates lipid peroxidation, resulting in the prevention of oxidative stress-related diseases^19,23–25^. Also, the extract of chia seeds alleviated neuroregeneration and several brain disorders, such as diabetic encephalopathy in diabetic rats^26^. It has been reported that the chia powder boosted antioxidant capacity, growth rate, and immunological status and decreased the stress-linked indicators ^27^.

Chia seeds oil (CSO) is a derivative of chia seeds^28,29^. CSO is a replacement source of omega-3-rich oil because of its high polyunsaturated fatty acids (PUFAs), principally ALA, linoleic acid, and oleic acids^30^. Previously, several studies have verified that CSO exhibited beneficial antioxidant, anti-inflammatory, antidepressant, memory enhancer, antimicrobial, anti-thrombotic and anti-arrhythmic, antidiabetic activity, as well as hypotensive and immunostimulatory effects^19,25,31^. In an attempt to gain a deeper insight into the probable significant effect of CSO in the management of chronic restraint-related alterations in rats, the expression of the corticotrophin-releasing factor (CRF) gene, brain's antioxidant and pro-inflammatory markers have been monitored. In addition, the histopathological, as well as immunohistochemical expression of apoptotic markers, inflammatory markers, and neurofunctional markers have been estimated. Furthermore, molecular docking studies were established.

## Material and methods

### Ethical declaration

The Ethics Committee of the Faculty of Medicine, Alexandria University, Egypt, issued the ethical approval serial code no. 0201545 for 2021. All trial protocols were achieved in concurrence with the Standard Guidelines for the Care and Use of Laboratory Animals and in accordance with ARRIVE guidelines. All ethical considerations and guidelines were followed during the testing period and specimen collection to ensure that animal suffering was effectively minimized.

### Chemicals

CSO (1000 mg/ capsule) was purchased as organic, unrefined, cold-pressed oil capsules (DEVA Nutrition LLC, USA, catalog number 63000), and the chemical composition of the investigated product was mentioned by the manufacturer^32^ and reported by Segura-Campos et al.^33^, Malko and Larraza^34^. Rat's serum corticosterone ELISA kit was purchased from ALPCO Co. (Keewaydin Drive, Salem, USA), serum tumor necrosis factor-alpha (TNF-α) and interleukin 6 (IL6) were purchased from Abcam (Cambridge, United Kingdom), and superoxide dismutase (SOD), catalase (CAT), glutathione peroxidase (GPx), and malondialdehyde (MDA) were obtained from Biodiagnostic Co. (Cairo, Egypt). All the chemicals and reagents used in this trial were of the finest quality and highest analytic grade.

### Laboratory animals

Fifty adult male Wister albino rats with an average age of 10–12 weeks and weight of 200 ± 20 g were used to conduct this trial. The animals were bought from the Faculty of Agriculture, Alexandria University, Egypt. All rats were maintained in an environment free from pathogens and housed in polypropylene cages in the Physiology Department laboratory, Faculty of Veterinary Medicine, Matrouh University. The laboratory's temperature was 19–22 °C. With relative humidity 60 ± 2% and a 12-h dark–light cycle were adjusted. All animals were given basic rodent pellets and filtered water ad libitum and were humanely handled and treated in agreement with the Authorized Animal Care Standards to ensure normal growth and behavior.

### Rat restrainer for immobilization and Stress paradigm

The rat restrainers used in this experiment were made of transparent plastic tubes suitable for the animal size and enough to initiate stress with minimum pain. One end of the tube had a conical tip with a breathing opening. The rat was inserted inside the strainer, with its head in the conical apex. Along the rest of the restrainer tube, there are perforating holes to allow ventilation. The rats were entirely controlled by stuffing the back end of the tube and securely shutting it with a stopper. To prevent animal habituation to the daily stress protocol, restraint times were regularly shifted during distinct periods of time^35^.

### Experiment design

**Figure 1:**
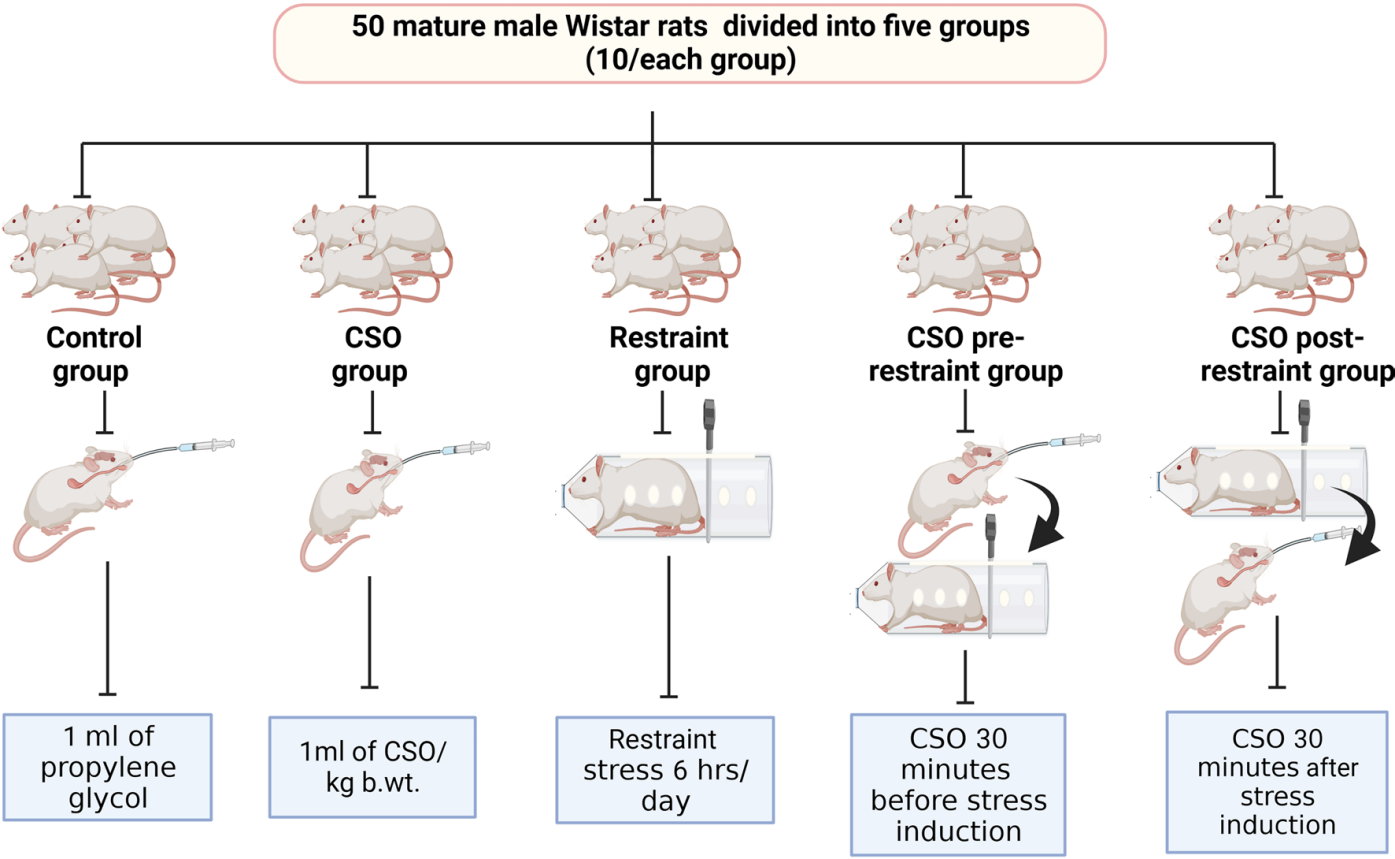
Diagram illustrates all the experimental groups and treatments.

After 2 weeks of acclimatization, fifty adult male Wistar albino rats were separated into five groups (10 rats each). Rats in the first group received 1 ml of propylene glycol/ kg BW/ day by gastric intubation and served as control. In the second group (CSO), rats in this group administrated 1 ml of CSO/kg b.wt. (equivalent to 400 mg of ALA/kg b.wt^36^ dissolved in propylene glycol)^37^. In the third group (restraint), rats were subjected to restraint stress 6 h/day by a rat strainer^35^. The fourth group (CSO pre-restraint) was administered 1 ml of CSO 30 min before immobilization stress. In the fifth group (CSO post-restraint), rats were administered 1 ml of CSO 30 min after immobilization stress. All the experimental groups and treatments were illustrated diagrammatically in Fig. 1.

All treatments were administrated daily for 60 successive days by gastric intubation. The CSO was freshly dissolved in propylene glycol to avoid the PUFAs oxidative deterioration. The rats were weighed once weekly during the experimental period using a digital balance in the morning.

### Blood and tissue sampling

At the experimental protocol end, Anahal (isoflurane 100%) was used for rat anesthesia for blood sampling and then euthanized. Twelve hrs. after the last session of stress, all groups were sacrificed. In order to collect serum via cardiac puncture, blood samples were collected and placed in sterile tubes without anticoagulant. The serum samples were centrifugated and preserved at −80 °C for later use. Brain tissues were immediately collected and divided longitudinally into two segments. The first segment was well-preserved at −80 °C for the additional antioxidant assessment in brain homogenate and gene expression analysis. The second segment was maintained in neutral buffered formaldehyde at 4 °C for 48 h for the additional histopathologic and immunohistochemical investigation.

### Assessment of the serum corticosterone levels

Rat's serum corticosterone concentrations were assessed by a commercially accessible ELISA kit (Cat. 55-CORMS-E01, ALPCO Co.) with an analytical sensitivity of 6.1 ng/ml based on the principle of competitive binding^38^. The corticosterone concentration was inversely proportional to the optical density estimated utilizing a microplate ELISA reader (Sorin Biomedica SpA., Milan, Lombardy, Italy) at 450 nm.

### Evaluation of oxidative/antioxidative markers in rats' brain homogenate

For the detection of superoxide dismutase (SOD), catalase (CAT), glutathione peroxidase (GPx), and malondialdehyde (MDA), immediately after dissection, the brain was rinsed in ice-cold phosphate buffer saline (PBS) solution pH 7.4, blotted with filter paper, weighed, and homogenized utilizing a tissue homogenizer in cold buffer (5 ml per gram tissue). The activity of GPx was evaluated in the brain tissues as previously described by Paglia and Valentine^39^ utilizing available kits (Cat. GP 2524, Biodiagnostic Co.), the assay based on the recycling of oxidized glutathione reduced state by the enzyme glutathione reductase and the oxidation of NADPH to NADP+ which is associated by a reduction in absorbance at 340 nm. The levels of SOD were evaluated in tissues using the available kits (Cat. SD 25 21 Biodiagnostic Co.) according to Nishikimi, et al.^40^. The assay relied on the inhibition of nitroblue tetrazolium dye reduction through phenazine methosulphate enzyme at 560 nm for 5 min for control and sample at 25 °C. Moreover, the level of CAT was estimated via the laboratory-supplied kits (Cat. CA 2517, Biodiagnostic Co.) based on the method detailed by Aebi^41^ by interaction with H_2_O_2_, and then inhibition of this reaction by CAT inhibitor at 510 nm. Estimation of MDA levels was performed using the commercially available kits (Cat. MD 2529, Biodiagnostic Co.) as formerly performed by Mihara and Uchiyama^42^. The assay was based on the reaction between thiobarbituric acid and MDA at the absorbance of 534 nm, resulting in a pink-colored complex.

### Estimation of serum pro-inflammatory mediators

Serum tumor necrosis factor-alpha (TNF-α; Cat. Ab208348, Abcam) and interleukin 6 (IL-6; Cat. Ab234570, Abcam) were colorimetrically identified by ELISA following the manufacturers' guidelines with analytical sensitivity 9.1 pg/ml and 43 pg/ml, respectively. The values of the relative optical density were performed utilizing a microplate ELISA reader (Sorin Biomedica SpA., Milan, Lombardy, Italy) at 450 nm.

### Analysis of CRF gene expression in the brain by quantitative polymerase chain reaction (qPCR)

The primer sequence of the CRF gene is shown in Table 1^43^. Utilizing QuantiTects Reverse Transcription Kit (Qiagen, USA), the total RNA (1 μg) reverse transcription into single-stranded complementary DNA (C-DNA) was performed in a two-step RT-PCR reaction. Real-time PCR was performed to evaluate mRNAs of CRF genes using Rotor-Gene Q (Qiagen, USA). By specific primers, the amplification of C-DNA amplicons was done by Maximas SYBR Green/Fluorescein qPCR Master Mix. The target gene relative expression was estimated using 2^−∆∆ct^, according to Livak and Schmittgen^44^. Relative gene expression fold change was estimated as follows: Fold changes = (2^−∆∆ct^).

**Table 1:** The primers’ sequence of CRF gene.

CRF	Forward primer	5′-AAATGGCCAGGGCAGAGCAGT-3′	^43^
	Reverse primer	5′-TGGCCAAGCGCAACATTTCAT-3′	
Housekeeping gene (GAPDH)	Forward primer	5′-CTACCCCCAATGTATCCGTTG-3′	
	Reverse primer	5′-AGCCCAGGATGCCCTTTAGT-3′	

### Histopathological examination

After anesthetizing by Anahal (isoflurane 100%), the animals were physically euthanized via cervical dislocation. The brain was extirped, rinsed out with PBS (pH 7.4), and immersed for 48 h in neutral buffered formaldehyde at 4 °C. The samples' fixing process was prepared by the paraffin embedding technique. Then, the staining of 4 µm thick sections was performed via Hematoxylin and Eosin (H and E) based on Bancroft and Layton^45^. The calculation of semiquantitative scoring of brain necrosis was based on the method of Gibson-Corley, et al.^46^. In brief, lesions from 10 fields of each rat were randomly selected from obtained micrographs from five rats and scored in a blinded way by a non-biased pathologist [score scale: 4 = 76–100%; 3 = 51–75%; 2 = 26–50%; 1 ≤ 25%; 0 = normal] then the scores were averaged.

### Immunohistochemistry (IHC)

Antibodies, sources, antigen retrieval methods, and working dilutions are listed in Table 2. The investigation of the immunohistochemical assay was based on Noreldin et al.^47^. To obtain micrographs, a digital camera (Leica EC3, Leica, Germany) linked to a Leica DM500 microscope was employed. Immunostaining area percentage was counted utilizing Image J software (National Institutes of Health, Bethesda, MD, USA)^48^ in ten randomly chosen areas obtained from each rat of the five rats in each group.

**Table 2:** Antibodies, sources, working dilutions, and antigen retrieval methods.

Antibody	Source	Dilution	Heating condition	Antigen retrieval
Polyclonal rabbit anti-cleaved Caspase-3	BioCare Medical, Cat. CP229C, Concord, CA, USA	1:100	–	–
Monoclonal rabbit anti-Cox-2	ThermoFisher Scientific, Cat: RM-9121-S0, Fremont, CA, USA	1:100	105 °C, 20 min	10 mM citrate buffer (pH 6.0)
Rabbit polyclonal anti-NFkB	ab31481, Abcam, Cambridge, UK	1:300	***–***	***–***
Mouse monoclonal anti- Il-6	Sc-57315, Santa Cruz, CA, USA	1:50	–	–
Rabbit polyclonal anti-Iba1	019-19741, Wako Osaka, Japan	1:1200	105 °C, 20 min	10 mM citrate buffer (pH 6.0)
Rabbit polyclonal anti-calbindin antibody	E10340, Spring Bioscience, Pleasanton, CA, USA	1:500	105 °C, 20 min	10 mM citrate buffer (pH 6.0)
Mouse monoclonal anti-synaptophysin	M7315, Dako, Glostrup, Denmark	1:50	105 °C, 20 min	10 mM citrate buffer (pH 6.0)
Rabbit polyclonal anti-GFAP antibody	ab7260, Abcam, Cambridge, UK	1:200	105 °C, 20 min	10 mM citrate buffer (pH 6.0)

### Molecular docking scores and interactions

The three-dimensional protein structures of caspase-3 (P55213), cyclooxygenase-2 (COX-2; P35355), corticotropin-releasing hormone binding protein (CRH-BP; P24388), corticotropin-releasing factor receptor 1 (CRHR1; P35353), corticotropin-releasing factor receptor 2 (CRHR2; P47866), interleukin-1 receptor type 1 (IL-1R1; Q02955), interleukin-1 receptor type 2 (IL-1R2; P43303), interleukin-6 receptor subunit alpha (IL6R; G3V8T6), interleukin-6 receptor subunit beta (IL6ST; P40190), and superfamily member 1A of tumor necrosis factor receptor (TNFRSF1A; P22934) were retrieved from and AlphaFold Protein Structure Database^49,50^. Furthermore, the three-dimensional structures of the active ingredient CSO were retrieved from the PubChem database^51,52^. The molecular docking was done using Molecular Operating Environment (MOE 2015.10) software after preparing target proteins and ligands. Finally, visualization of ligands-proteins interactions was determined with Discovery Studio Visualizer software tool^53^.

### Statistical analysis

To analyze the laboratory data statistically, a Statistical Program for the Social Science Software version 22 (SPSS Inc., Chicago, IL) was performed. To determine the normality of the testing results for the animals, the Kolmogorov–Smirnov technique was used^54,55^. One-way analysis of variance (ANOVA) was conducted for all variables^56^. Values are denoted as the mean ± standard error, and then Tukey's posthoc analysis is performed^57^. The overall significance was recognized at 0.05. Correlations among brain CRF gene expression, serum corticosterone, proinflammatory cytokines, brain oxidative stress markers, and immunoexpression were studied using the Pearson correlation coefficient of pairwise comparison^58^. The correlation heatmap was used to identify these correlations between samples obtained by OriginPro, Version 2023b. (OriginLab Corporation, Northampton, MA, USA). Furthermore, the percentage change = [(mean of the control group − mean of the treated group)/mean of control group] × 100%, the percentage improvement = [(mean of the stressed group − mean of the treated group)/mean of control group] × 100^59^.

## Results

### Assessment of serum corticosterone

As displayed in Table 3, the restrained group has a significant (P < 0.05) elevation in the corticosterone level related to the control and CSO groups. However, the CSO pre-restraint and CSO post-restraint groups displayed a significant (P < 0.05) decline in their serum corticosterone level compared to the stressed group. The CSO-treated rats displayed a non-significant (P < 0.05) difference relative to the control rat, as well as there was a non-statistical (P < 0.05) variance between the pre and post-restraint groups.

**Table 3:** Effect of chronic restraint stress, chia seed oils (CSO), or their combination on serum corticosterone, oxidative stress markers (GPx, CAT, SOD, MDA), proinflammatory markers (TNF-α and IL-6), and CRF gene expression in the brain of mature male rats.

Group	Control	CSO	Restraint	CSO pre-restraint	CSO post-restraint
Parameter					
Corticosterone (ng/ml) % change % improvement	273.67 ± 3.05^c^ – –	290.38 ± 3.87^c^ −5.8% –	441.88 ± 4.46^a^ −60% –	316.08 ± 5.42^b^ −15.3% 45.9%	327.76 ± 4.61^b^ −19.6% 41.6%
GPx (U/g tissue) % change % improvement	42.56 ± 0.82^a^ – –	44.39 ± 3.5^a^ −4.3% –	22.33 ± 1.86^b^ 47.5% –	38.28 ± 1.83^a^ 10.1% 37.5%	36.35 ± 1.02^a^ 14.6% 32.9%
CAT (U/g tissue) % change % improvement	36.26 ± 1.21^a^ – –	36.5 ± 1.62^a^ −0.7% –	15.49 ± 0.89^c^ 57.3% –	26.47 ± 1.21^b^ 26.9% 30.3%	22.05 ± 0.96^b^ 39.2% 18.1%
SOD (U/g tissue) % change % improvement	66.48 ± 1.49^a^ – –	64.65 ± 0.36^a^ 2.8% –	31.39 ± 1.22^d^ 52.8% –	53.4 ± 1.78^b^ 19.7% 33.1%	39.48 ± 1.15^c^ 40.6% 12.2%
MDA (nmol/g tissue) % change % improvement	4.99 ± 0.19^c^ – –	4.09 ± 0.25^c^ 18% –	14.77 ± 0.57^a^ −195.9% –	8.19 ± 0.27^b^ −64.1% 131.9%	9.31 ± 0.25^b^ −86.6% 109.4%
IL-6 (pg/ml) % change % improvement	3.96 ± 0.28^c^ – –	3.75 ± 0.22^c^ 5.3% –	18 ± 1.64^a^ −354% –	6 ± 1.11^bc^ −51.5% 303%	9.66 ± 1.24^b^ −143.9% 210%
TNF-α (pg/ml) % change % improvement	47.08 ± 2.28^d^ – –	45.54 ± 1.53^d^ 0.3% –	122.15 ± 3.08^a^ −159.5% –	75.52 ± 2.83^c^ −60.4 99%	98.65 ± 2.26^b^ −109% 49.9%
Expression fold change of CRF gene % change % improvement	0.36 ± 0.02^d^ – –	0.37 ± 0.03^d^ −2.7% –	1.1 ± 0.04^a^ −205% –	0.67 ± 0.02^c^ −86% 119%	0.87 ± 0.02^b^ −142% 63.8%

All data are presented as means ± S.E. (n = 10). Means with different letters (a, b, c, d) within the same column are significantly different at a p-value < 0.05.

*CSO* chia seeds oil, *GPx* glutathione peroxidase, *CAT* catalase, *SOD* superoxide dismutase, *MDA* malondialdehyde, *IL-6* interleukin-6, *TNF-α* tumor necrosis factor-alpha, *CRF* corticotrophin-releasing factor.

### Evaluation of oxidative/antioxidative regulators

The oxidative/antioxidative regulators of brain tissues were evaluated and presented in Table 3. The GPx, CAT, and SOD levels were markedly (P < 0.05) lowered in the stressed group in comparison to all other groups (control, CSO, CSO pre-restraint, and CSO post-restraint). In particular, GPx exhibited a non-significant (P < 0.05) variation between the control, CSO, CSO pre-restraint, and CSO post-restraint groups.

On the other hand, the lipid peroxidation marker (MDA) displayed a marked (P < 0.05) elevation in its level in the stressed group compared to all other groups (control, CSO, CSO pre-restraint, and CSO post-restraint). While in the CSO pre-restraint and CSO post-restraint groups, the levels of MDA were markedly (P < 0.05) lowered in comparison to the stressed group.

### Estimation of pro-inflammatory cytokines

As noted in Table 3, a marked (P < 0.05) increase in the level of IL-6 and TNFα was detected in the restrained group relative to all other rat groups (control, CSO, CSO pre-restraint, and CSO post-restraint). Specifically, the CSO pre-restraint group did not show any marked (P < 0.05) difference in IL-6 relative to the control and CSO groups. While the levels of TNFα in the CSO pre-restraint group were markedly (P < 0.05) decreased relative to the CSO post-restraint group. No significant variance was detected between the control and CSO groups in both IL-6 and TNFα.

### Analysis of CRF gene expression

Data represented in Table 3 showed that the CRF gene expression in the brain tissues was markedly (P < 0.05) elevated in the stressed group (restraint) as compared to their counterparts in all other groups (control, CSO, CSO pre-restraint and CSO post-restraint). Interestingly, the CSO pre-restraint group shows a statistical (P < 0.05) reduction in the expression of the CRF gene compared to the CSO post-restraint.

### Histopathologic examination

**Figure 2:**
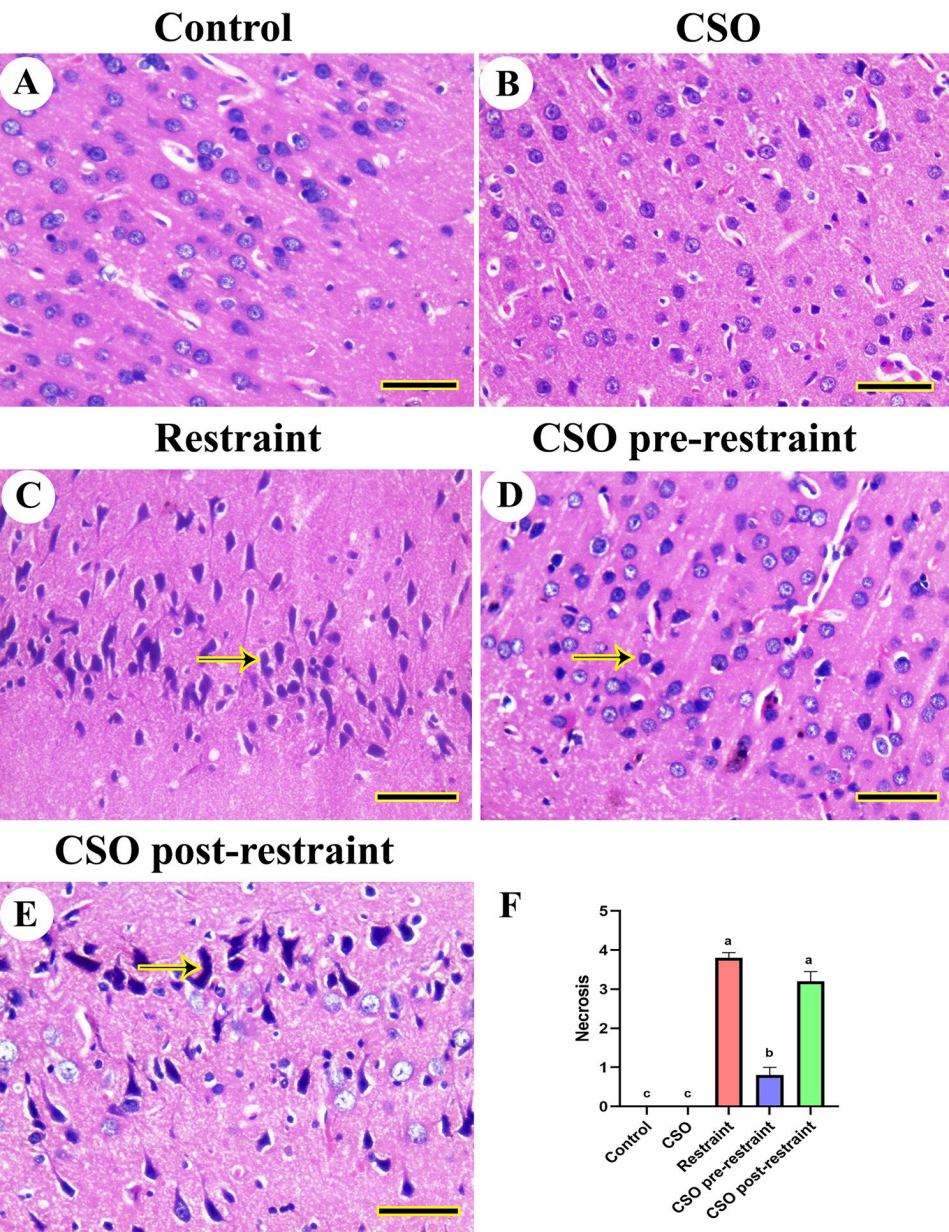
Representative photomicrograph (HE, scale bar = 50 µm) of cerebrum tissues from rats subjected to chronic immobilization stress and/or chia seeds oil (CSO) pre and post-restraint: (**A**) Control, and (**B**) CSO-treated rats displaying normal neuroarchitecture. (**C**) Restraint-exposed rats showing darkly necrotic shrunken neurons (arrow). (**D**) CSO pre-restraint rats reveal the nearly normal architecture of neuropil and neurons. (**E**) CSO post-restraint rats showing apparently normal cerebral architecture with some necrotic neurons (arrow). (**F**) Semiquantitative histomorphometric evaluation of neuronal necrosis. All values are expressed as the mean ± SEM.

The histopathologic investigation of the cerebrum of control and CSO-treated rats revealed a normal histologic picture of the neuropil and neurons (Fig. 2A, B). On the contrary, the cerebral tissues of restrained rats showed necrotic neurons (Fig. 2C). However, the cerebrum of CSO pre-restrained rats revealed quite normal cerebral architecture (Fig. 2D). Moreover, CSO post-restrained rats have revealed an improvement in neuropil and cerebrum neurons with minimal necrosis of neurons (Fig. 2E).

**Figure 3:**
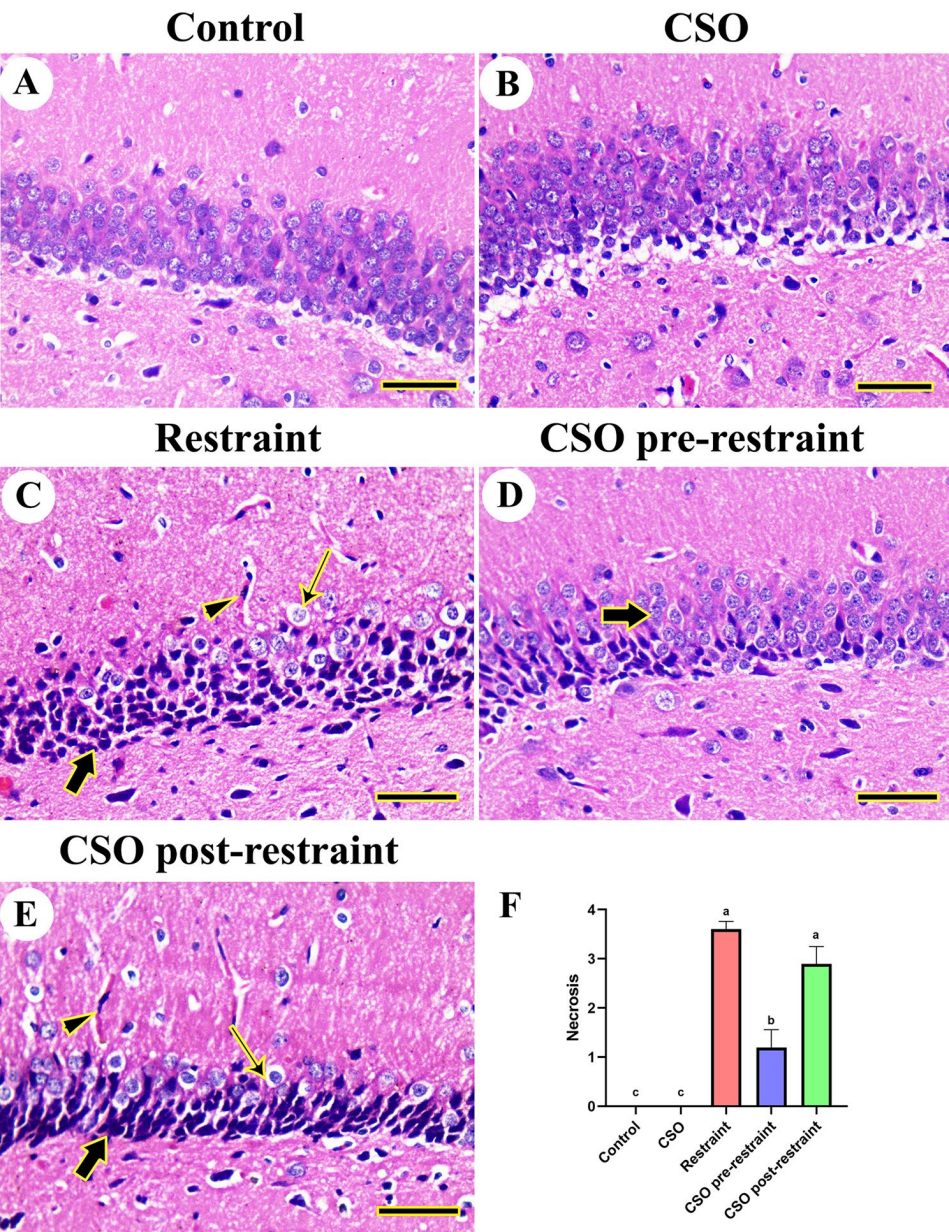
Representative photomicrograph (HE, scale bar = 50 µm) of hippocampal tissues from rats exposed to chronic immobilization stress and/or chia seeds oil (CSO) pre and post-restraint: (**A**) Control, and (**B**) CSO-treated rats revealed a normal histologic picture of the dentate gyrus. (**C**) restraint-exposed rats exposing congested blood vessels (arrowhead), shrunken neurons (thick arrow), and hyperchromatic neurons (thin arrow). CSO pre-restraint rats (**D**) and CSO post-restraint rats (**E**) revealed apparently normal hippocampal architecture with mild neuronal degeneration. (**F**) Semiquantitative histomorphometric evaluation of neuronal necrosis. All values are expressed as the mean ± SEM.

The histopathologic evaluation for the neural tissues obtained from the hippocampus in control and CSO-treated groups revealed the normal structure of the dentate gyrus molecular and granule cell layer (Fig. 3A, B). However, chronically restrained rats displayed necrosis, disordered arrangement, and decreased numbers of dentate gyrus neurons (Fig. 3C). Rats in CSO pre-restraint and CSO post-restraint groups revealed improved morphology of dentate gyrus, with mild neuronal degeneration (Fig. 3D, E).

**Figure 4:**
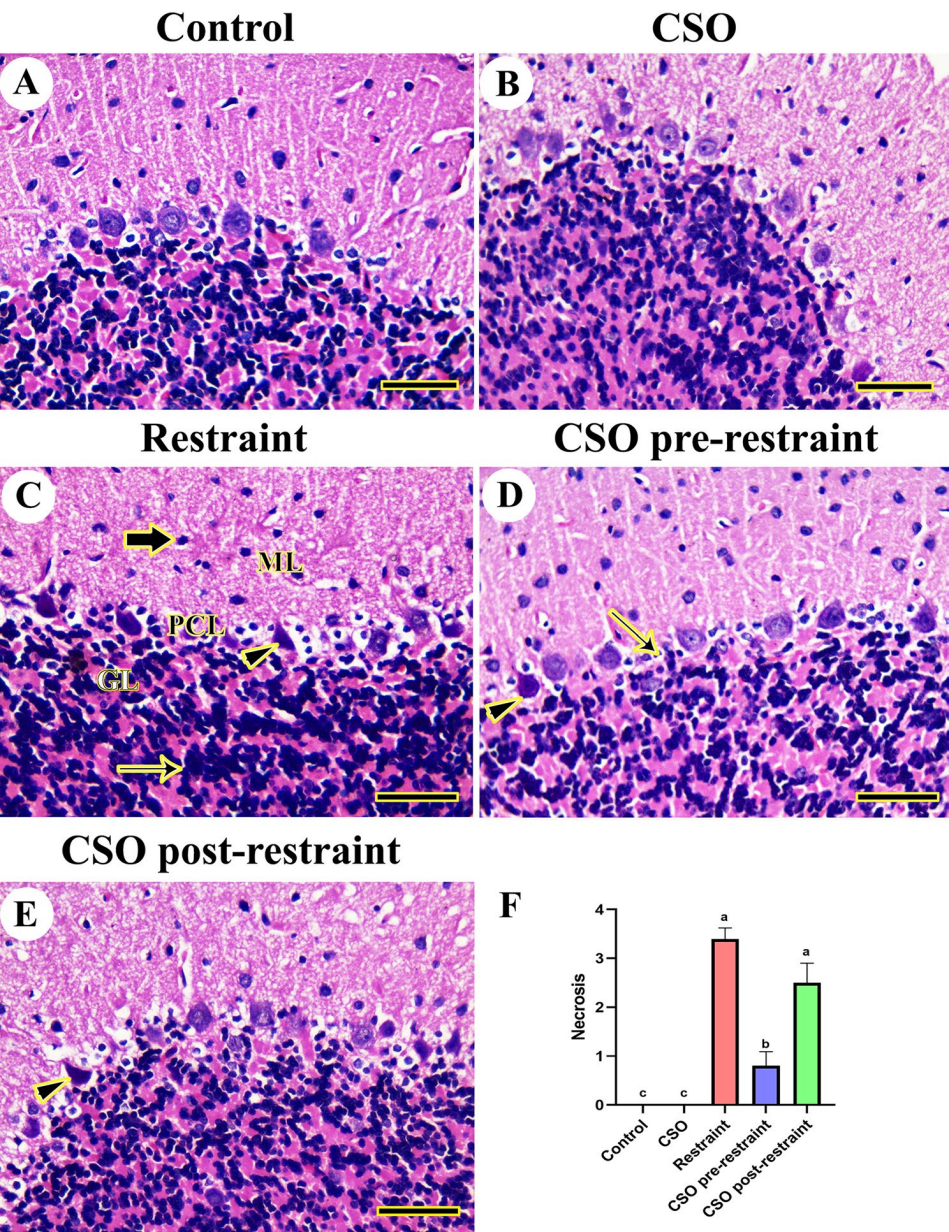
Representative photomicrograph (HE, scale bar = 50 µm) of cerebellum tissues from rats exposed to chronic immobilization stress and/or chia seeds oil (CSO) pre and post-restraint: (**A**) control, and (**B**) CSO-treated rats showing a normal cerebellar neuroarchitecture. (**C**) Restraint-exposed rats displaying shrunken neurons (thick arrow) in the molecular layer (ML), hypertrophic neurons in the granule cell layer (thin arrow) in the granular layer (GL), and shrunken Purkinje cells (arrowhead) distributed within Purkinje cells layer (PCL). (**D**) CSO pre-restraint rats exposing semi-normal histoarchitecture with a pyknotic (arrowhead) or few lost (arrow) Purkinje cells. (**E**) CSO post-restraint rats showed normal hippocampal histoarchitecture with more degenerated (arrowhead) Purkinje cells than CSO post-restraint rats. (**F**) Semiquantitative histomorphometric evaluation of neuronal necrosis. All values are expressed as the mean ± SEM.

Upon the examination of rats' cerebellum in control and CSO-treated groups, normal histological limits of molecular, granular, and Purkinje layers were detected in the cerebellar areas (Fig. 4A, B). However, rats in the restraint group showed focal neuronal loss of the granular cell layer with a complete nuclei loss in the necrotic Purkinje layer (Fig. 4C). However, CSO pre-restrained rats displayed semi-typical histological architecture of the cerebellar region with minimum degenerated Purkinje cells (Fig. 4D). Furthermore, rats in the CSO post-restraint group showed a better histologic picture of the cerebellum with mild pyknosis of Purkinje cells than the restraint group only (Fig. 4E).

Results from the semiquantitative scoring for brain necrosis in the cerebral, cerebellar, and hippocampal tissues confirmed a significant (P < 0.05) elevation in neuronal necrosis scores in all brain regions of restrained rats compared to control rats. However, rats in the CSO pre-restraint and CSO post-restraint groups demonstrated a marked (P < 0.05) decrease in neuronal necrosis scoring in different brain regions relative to their counterparts in the restraint group (Figs. 2F, 3F, and 4F).

### Immunohistochemical expression

#### Caspase-3 

**Figure 5:**
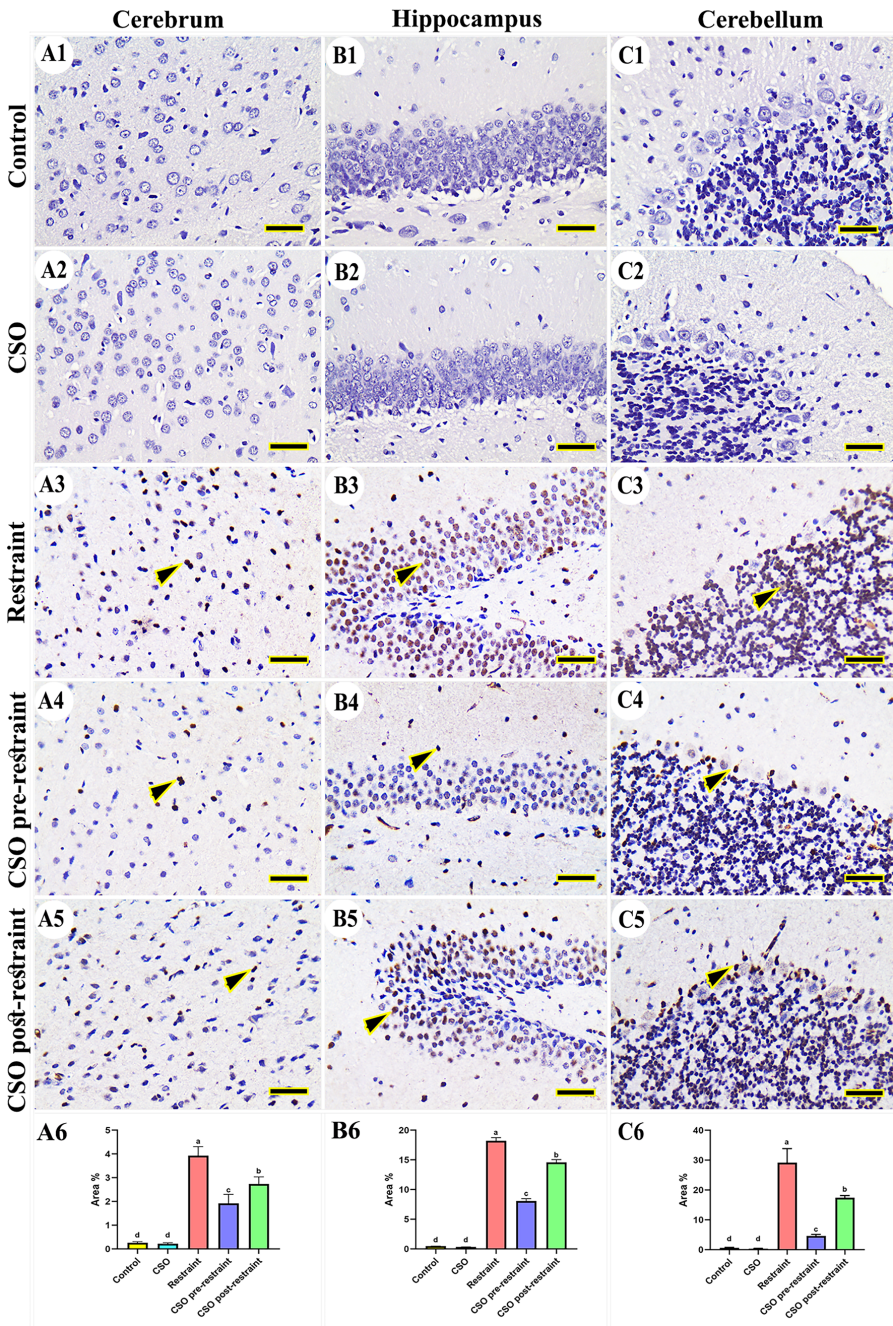
Representative photomicrograph (scale bar = 50 µm) for immunoreactivity of Caspase-3 in cerebral (**A1–A5**), hippocampal (**B1–B5**), and cerebellar (**C1–C5**) from control (**A1, B1, C1**), CSO (**A2, B2, C2**), restraint (**A3, B3, C3**), CSO pre-restraint (**A4, B4, C4**), and CSO post-restraint (**A5, B5, C5**). Arrowheads indicate positive immune expression in control and treated groups. Statistical analysis for area % of caspase-3 immunoexpression in the cerebral (**A6**), hippocampal (**B6**), and cerebellar (**C6**) of control and treated groups. All values are expressed as the mean ± SEM. *CSO* chia seeds oil.

The investigation of caspase-3 immunohistochemical reaction in control and CSO groups revealed negative immune expression in the cerebral (Fig. 5A1 and A2), hippocampal (Fig. 5B1 and B2), and cerebellar (Fig. 5C1 and C2) tissues, respectively. However, the restraint group showed an increased number of caspase-3 immune-positive nuclei in different regions of the brain (Fig. 5A3, B3, and C3). On the other hand, the CSO pre-restrained group showed the most minimal distribution of caspase-3 positive reacted nucleus (Fig. 5A4, B4, and C4). Moreover, the group treated with CSO post-restraint exhibited a low caspase-3 distribution (Fig. 5 A5, B5, C5). The statistical evaluation for the area % of caspase-3 showed high expression (P < 0.05) in the restraint group in comparison to the control and CSO rat groups. The detected expression declined markedly (P < 0.05) in the CSO pre-restraint and CSO post-restraint groups compared to restrained rats (Fig. 5A6, B6, C6).

#### Cyclooxgynase-2 (COX-2)

**Figure 6:**
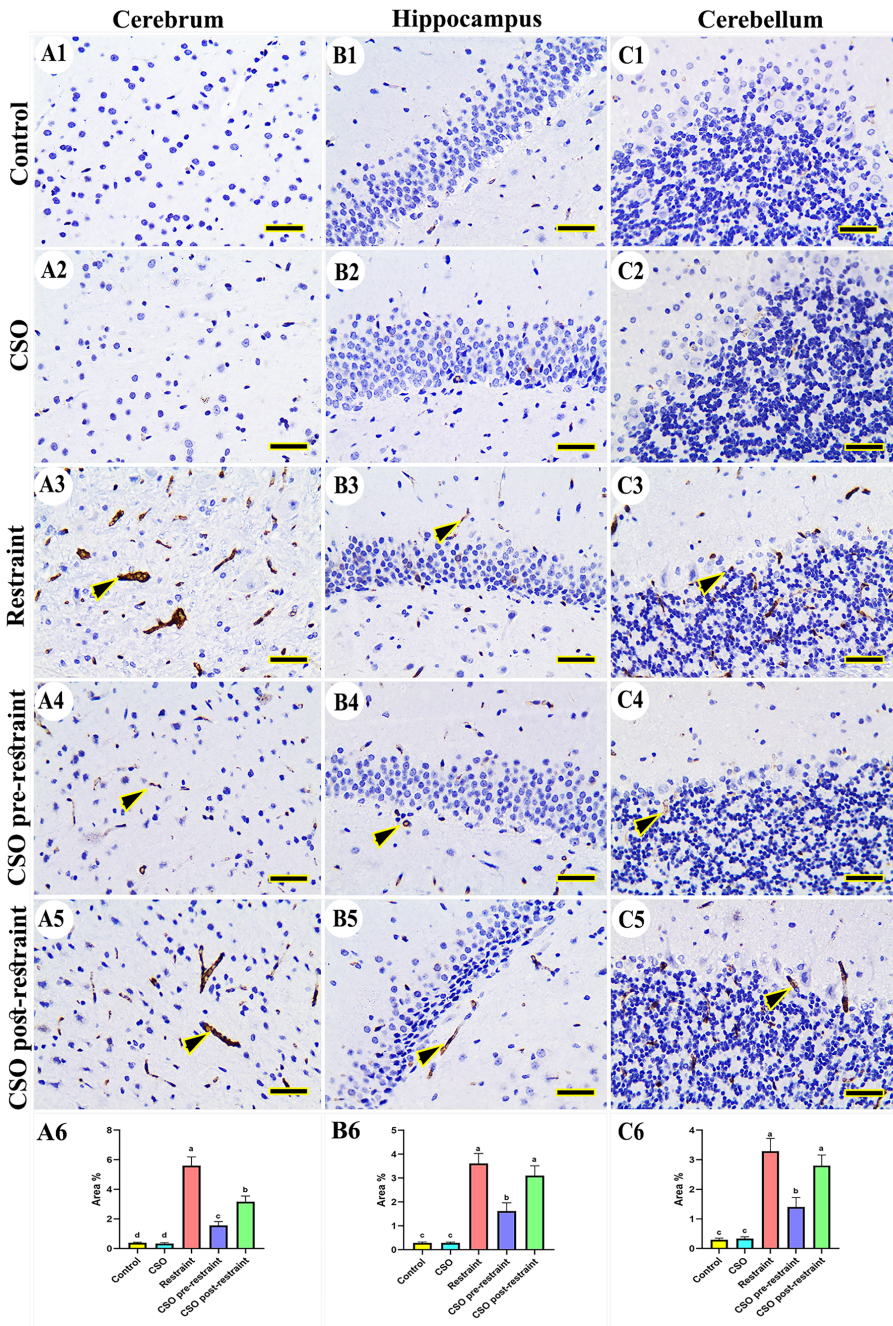
Representative photomicrograph (scale bar = 50 µm) for immunoreactivity of Cyclooxgynase-2 (COX-2) in cerebral (**A1–A5**), hippocampal (**B1–B5**), and cerebellar (**C1–C5**) from control (**A1, B1, C1**), CSO (**A2, B2, C2**), restraint (**A3, B3, C3**), CSO pre-restraint (**A4, B4, C4**), and CSO post-restraint (**A5, B5, C5**). Arrowheads indicate positive immune expression in control and treated groups. Statistical analysis for area % of COX-2 immunoexpression in the cerebral (**A6**), hippocampal (**B6**), and cerebellar (**C6**) of control and treated groups. All values are expressed as the mean ± SEM. *CSO* chia seeds oil.

In the control and CSO groups, there was no expression for COX-2 to be noticed in the cerebral (Fig. 6A1, A2), hippocampal (Fig. 6B1, B2), and cerebellar tissues (Fig. 6C1, C2). Alternatively, the rats exposed to chronic restraint stress revealed marked reaction for COX-2 in different brain areas (Fig. 6A3, B3, C3), which was lowered considerably (P < 0.05) in the samples isolated from the CSO pre-restraint group (Fig. 6A4, B4, C4). The COX-2 immuno-expression was low (P < 0.05) in the CSO post-restrained rats (Fig. 6A5, B5, C5). The statistical analysis for the COX-2 area percentage showed marked (P < 0.05) COX-2 immune reaction in the stressed rats relative to control and CSO groups. This immuno-expression was markedly (P < 0.05) lowered in the CSO pre-restraint and CSO post-restraint groups (Fig. 6A6, B6, C6).

#### Nuclear factor kappa B (NFkB)

**Figure 7:**
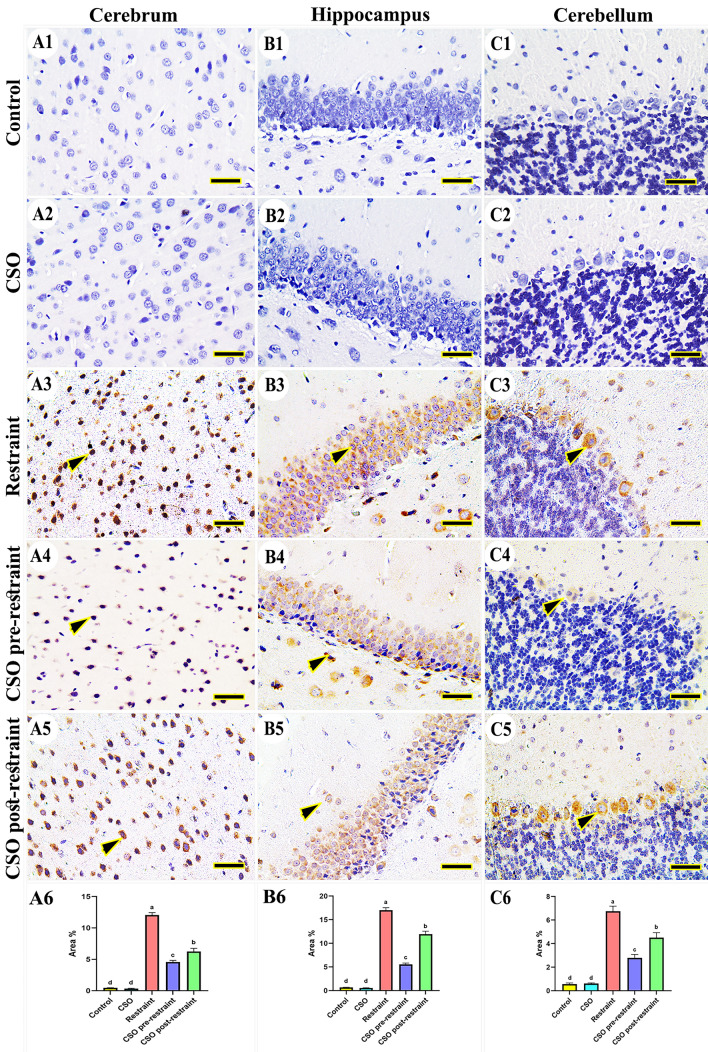
Representative photomicrograph (scale bar = 50 µm) for immunoreactivity of nuclear factor kappa B (NFkB) in the cerebral (**A1–A5**), hippocampal (**B1–B5**), and cerebellar (**C1–C5**) from control (**A1, B1, C1**), CSO (**A2, B2, C2**), restraint (**A3, B3, C3**), CSO pre-restraint (**A4, B4, C4**), and CSO post-restraint (**A5, B5, C5**). Arrowheads indicate positive immune expression in control and treated groups. Statistical analysis for area % of NFkB immunoexpression in the cerebral (**A6**), hippocampal (**B6**), and cerebellar (**C6**) of control and treated groups. All values are expressed as the mean ± SEM. *CSO* chia seeds oil.

In the control and CSO groups, there was no expression for NF-kB noticed in the cerebral (Fig. 7A1, A2), hippocampal (Fig. 7B1, B2), and cerebellar tissues (Fig. 7C1, C2). On the contrary, the rats subjected to restraint stress revealed marked expression of NFkB in all brain areas (Fig. 7A3, B3, C3), which markedly decreased in the tissues collected from rats in the CSO pre-restraint group (Fig. 7A4, B4, C4). The NFkB expression was low in the CSO post-restraint group (Fig. 7A5, B5, C5). The statistical analysis for the NFkB area % revealed an extensive (P < 0.05) immune reaction of NFkB in the stressed rats compared to the control and CSO groups. Marked (P < 0.05) reduction in this reaction was detected in the CSO pre-restraint and CSO post-restraint groups (Fig. 7A6, B6, C6).

#### Interleukin 6 (IL-6)

**Figure 8:**
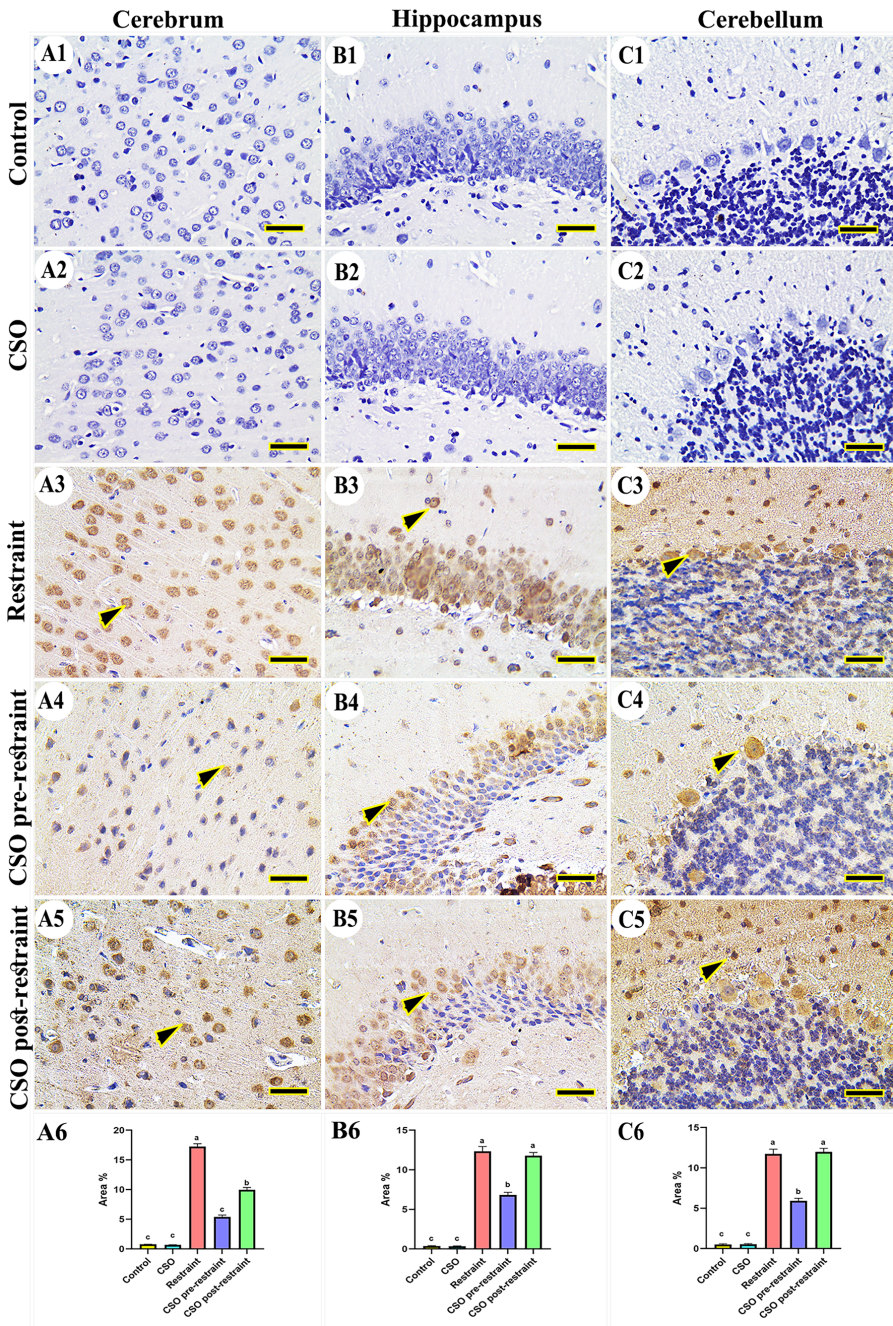
Representative photomicrograph (scale bar = 50 µm) for immunoreactivity of interleulin-6 (IL-6) in cerebral (**A1–A5**), hippocampal (**B1–B5**), and cerebellar (**C1–C5**) from control (**A1, B1, C1**), CSO (**A2, B2, C2**), restraint (**A3, B3, C3**), CSO pre-restraint (**A4, B4, C4**), and CSO post-restraint (**A5, B5, C5**). Arrowheads indicate positive immune expression in control and treated groups. Statistical analysis for area % of IL-6 immunoexpression in the cerebral (**A6**), hippocampal (**B6**), and cerebellar (**C6**) of control and treated groups. All values are expressed as the mean ± SEM. *CSO* chia seeds oil.

Concerning the study of IL-6 expression in the control and CSO rats, no immunoreactivity was noticed in cerebral (Fig. 8A1, A2), hippocampal (Fig. 8B1, B2), and cerebellar regions (Fig. 8C1, C2). On the other hand, the rats subjected to chronic immobilization revealed marked immune expression of IL-6 in all regions of the brain (Fig. 8A3, B3, C3); this reaction was markedly decreased in the brain tissues collected from the CSO pre-restraint group (Fig. 8A4, B4, C4). The immunoreactivity of IL-6 was low in the CSO post-restraint group (Fig. 8A5, B5, C5). The statistical analysis for the IL-6 area % revealed a marked (P < 0.05) increase in the restraint group in comparison to control and CSO rats. This reaction was markedly (P < 0.05) decreased in the CSO pre-restraint and CSO post-restraint groups (Fig. 8A6, B6, C6).

#### Calbindin-28K

**Figure 9:**
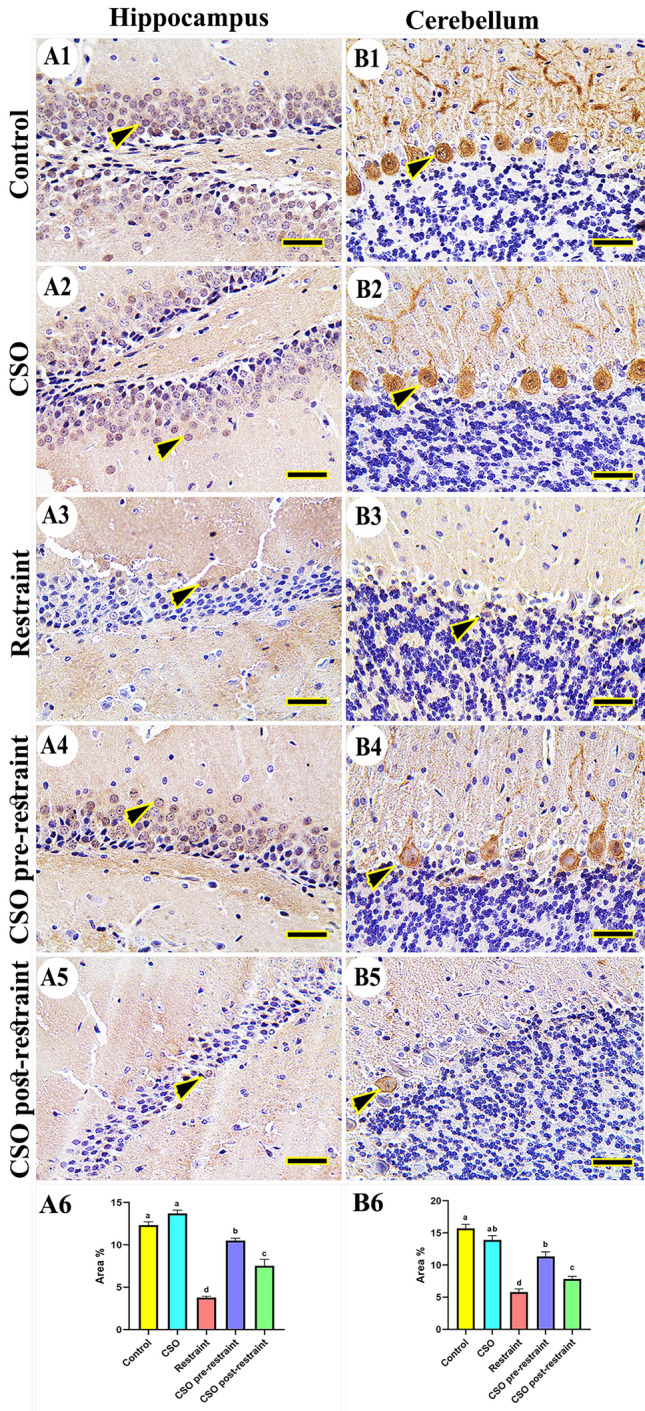
Representative photomicrograph (scale bar = 50 µm) for immunoreactivity of Calbindin-28K in hippocampal (**A1–A5**) and cerebellar (**B1–B5**) from control (**A1, B1**), CSO (**A2, B2**), restraint (**A3, B3**), CSO pre-restraint (**A4, B4**), and CSO post-restraint (**A5, B5**). Arrowheads indicate positive immune expression in control and treated groups. Statistical analysis for area % of calbindin-28K immunoexpression in the hippocampal (**A6**) and cerebellar (**B6**) of control and treated groups. All values are expressed as the mean ± SEM. *CSO* chia seeds oil.

Concerning calbindin-28K immune reaction in control and CSO rats, although there was no immune expression in cerebral tissues of control and CSO-treated groups, increased immune expression was seen in the hippocampus (Fig. 9A1, A2) and cerebellum (Fig. 9B1, B2), respectively. However, the restrained rats showed the lowest expression in all brain compartments (Fig. 9A3, B3). On the other hand, calbindin-28K expression was obviously noted in the CSO pre-restraint group (Fig. 9A4, B4), followed by CSO post-restraint (Fig. 9A5, B5). The statistical analysis for the calbindin-28K area percentage showed an extensive (P < 0.05) reduction in calbindin-28K expression in the restrained rats in comparison to control and CSO rats. The detected reaction was extensively (P < 0.05) increased in the CSO pre-restraint and CSO post-restraint groups (Fig. 9A6, B6).

#### Ionized calcium-binding adaptor molecule 1 (IBA1)

**Figure 10:**
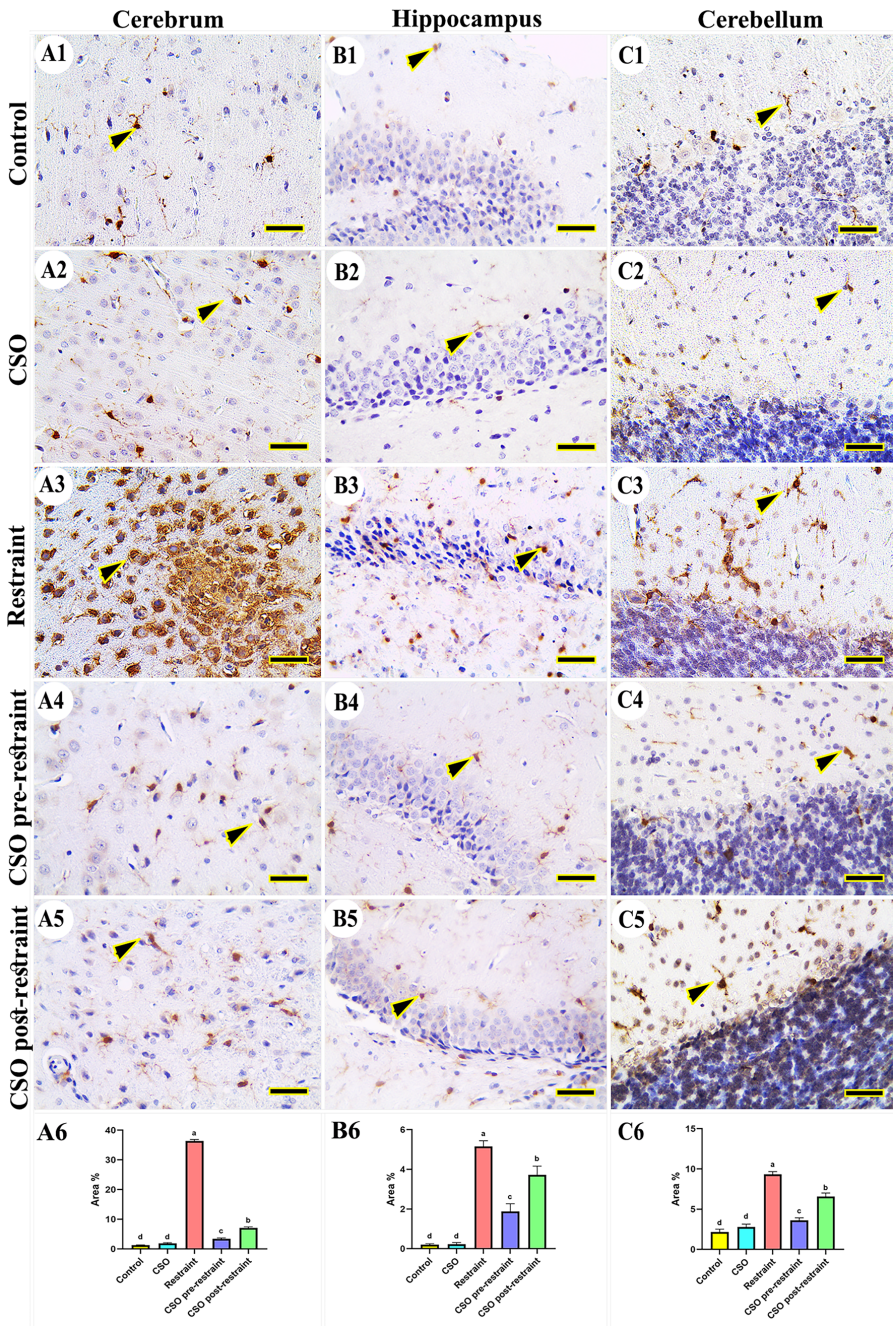
Representative photomicrograph (scale bar = 50 µm) for microglia distribution using immunoreactivity for IBA1 in cerebral (**A1–A5**), hippocampal (**B1–B5**), and cerebellar (**C1–C5**) from control (**A1, B1, C1**), CSO (**A2, B2, C2**), restraint (**A3, B3, C3**), CSO pre-restraint (**A4, B4, C4**), and CSO post- restraint (**A5, B5, C5**). Arrowheads indicate positive immune expression in control and treated groups. Statistical analysis for area % of IBA1 immunoexpression in the cerebral (**A6**), hippocampal (**B6**), and cerebellar (**C6**) of all control and treated groups. All values are expressed as the mean ± SEM. *CSO* chia seeds oil.

The detection of IBA1 immuno-expression in control and CSO rats showed the fewest microglia in the cerebral (Fig. 10A1, A2), hippocampal (Fig. 10B1, B2), and cerebellar tissues (Fig. 10C1, C2). Otherwise, rats in the restraint group showed marked distribution of microglia in different brain areas (Fig. 10A3, B3, C3). On the other hand, CSO pre-restraint treated rats displayed the fewest microglia number in all areas of the brain (Fig. 10A4, B4, C4). Furthermore, rats in the CSO post-restraint group had a lower microglia distribution (Fig. 10A5, B5, C5). The statistical analysis for the IBA1 area % revealed markedly (P < 0.05) high immunohistochemical reaction in the restrained group in comparison to the control and CSO groups. This reaction was statistically (P < 0.05) lowered in the CSO pre-restraint and CSO post-restraint groups (Fig. 106, B6, C6).

#### Synaptophysin (SYP)

**Figure 11:**
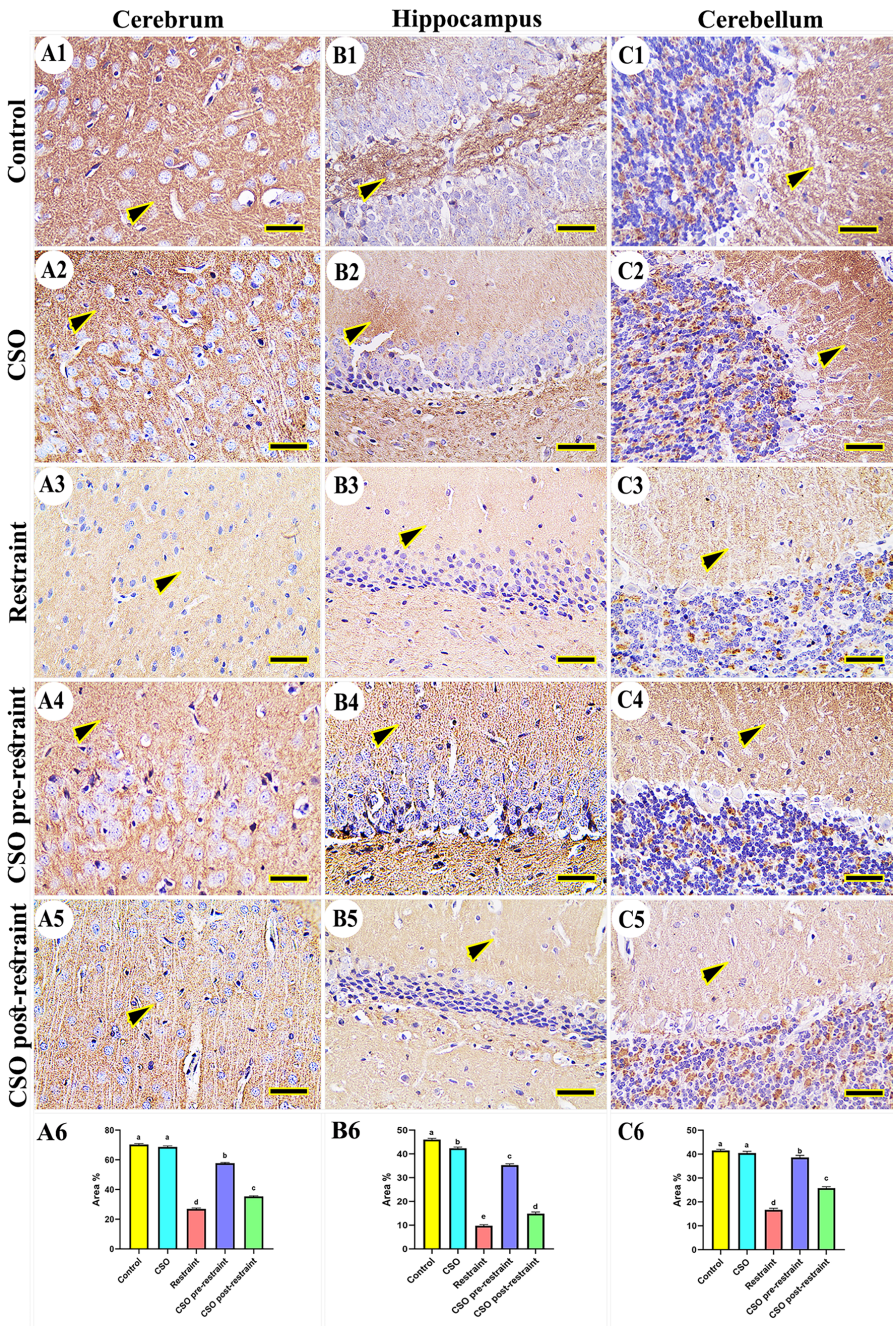
Representative photomicrograph (scale bar = 50 µm) for immunoreactivity of synaptophysin (SYP) in the cerebral (**A1–A5**), hippocampal (**B1–B5**), and cerebellar (**C1–C5**) from control (**A1, B1, C1**), CSO (**A2, B2, C2**), restraint (**A3, B3, C3**), CSO pre-restraint (**A4, B4, C4**), and CSO post-restraint (**A5, B5, C5**). Arrowheads indicate positive immune expression in control and treated groups. Statistical analysis for area % of SYP immunoexpression in the cerebral (**A6**), hippocampal (**B6**), and cerebellar (**C6**) of control and treated groups. All values are expressed as the mean ± SEM. *CSO* chia seeds oil.

The SYP expressions in the brain of rats from control and CSO groups were normal in the cerebral (Fig. 11A1, A2), hippocampal (Fig. 11B1, B2), and cerebellar tissues (Fig. 11C1, C2). On the contrary, the immunoreactivity of SYP was markedly decreased in the brain tissues collected from restrained rats (Fig. 11A3, B3, C3). While, marked improvement in the SYP immunoreactivity was noticed in rats of the CSO pre-restraint group (Fig. 11A4, B4, C4), followed by those in the CSO post-restraint group (Fig. 11A5, B5, C5). The statistical analysis for the SYP area percentage has shown a marked (P < 0.05) reduced SYP expression in the stressed rats in comparison with the control rats. This immunoreaction was markedly (P < 0.05) augmented in the CSO pre-restraint and CSO post-restraint groups (Fig. 11A6, B6, C6).

#### Glial fibrillary acidic protein (GFAP)

**Figure 12:**
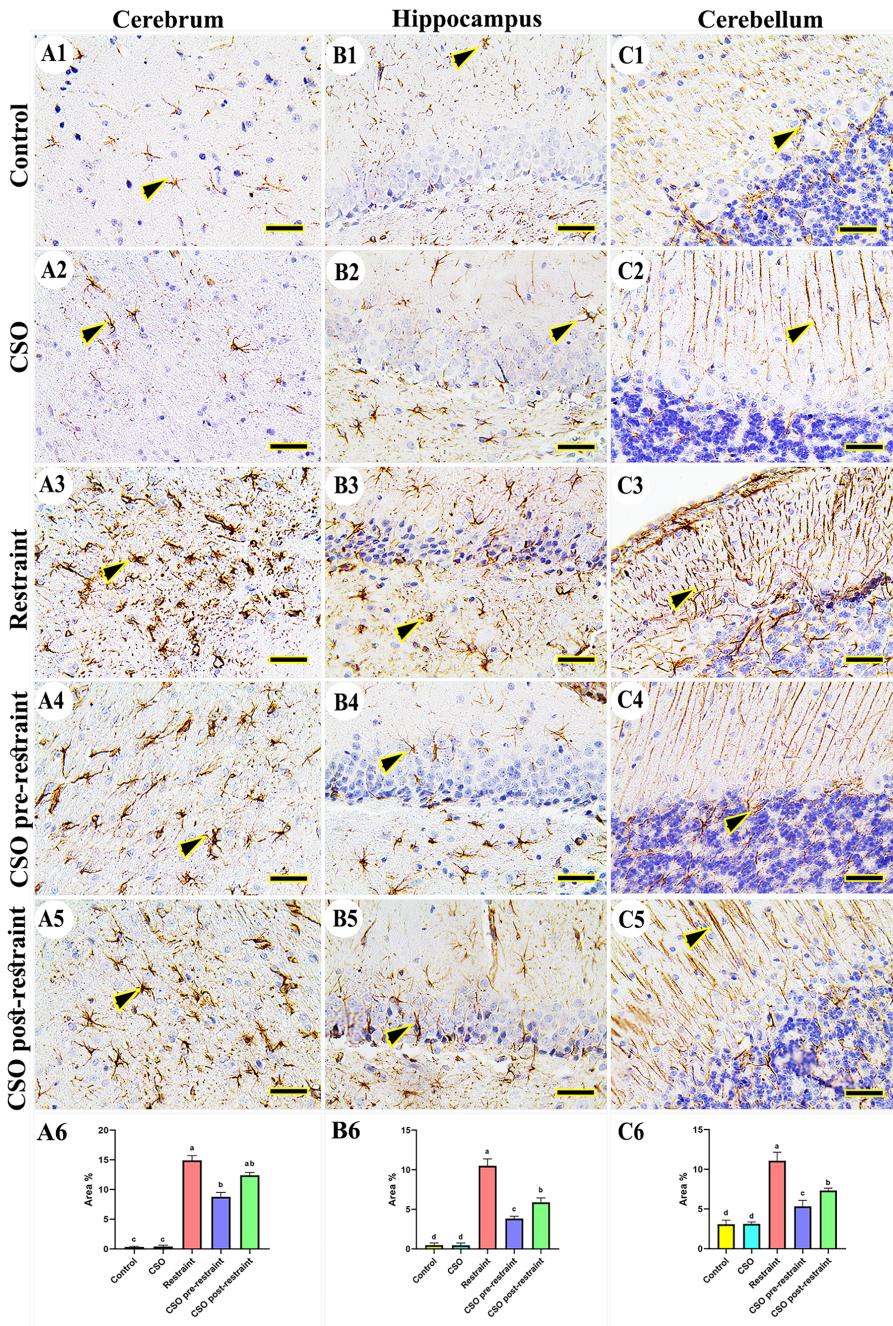
Representative photomicrograph (scale bar = 50 µm) for astrocytes distribution using immunoreactivity for GFAP in cerebral (**A1–A5**), hippocampal (**B1–B5**), and cerebellar (**C1–C5**) from control (**A1, B1, C1**), CSO (**A2, B2, C2**), restraint (**A3, B3, C3**), CSO pre-restraint (**A4, B4, C4**), and CSO post-restraint (**A5, B5, C5**). Arrowheads indicate positive immune expression in control and treated groups. Statistical analysis for area % of GFAB immunoexpression in the cerebral (**A6**), hippocampal (**B6**), and cerebellar (**C6**) of all control and treated rats. All values are expressed as the mean ± SEM. *CSO* chia seeds oil.

Regarding the GFAP immune reaction in rat brains in the control and CSO groups, reduced astrocytes distribution was detected in cerebral (Fig. 12A1, A2), hippocampal (Fig. 12B1, B2), and cerebellar tissues (Fig. 12C1, C2). Meanwhile, the restrained rats showed a marked number of astrocytes in all brain regions (Fig. 12A3, B3, C3). Rats in both CSO pre-restraint and CSO post-restraint showed few distributions of astrocytes in all areas of the brain (Fig. 12A4, B4, C4) and (Fig. 12A5, B5, C5) respectively. The statistical analysis for the GFAP area % in astrocytes showed extensive (P < 0.05) immuno-expression of GFAP in the immobilized rats than in control and CSO groups. This reaction was markedly (P < 0.05) lowered in the CSO pre-restraint and CSO post-restraint groups (Fig. 12A6, B6, C6) relative to restrained rats.

### Molecular docking scores and interactions

**Figure 13:**
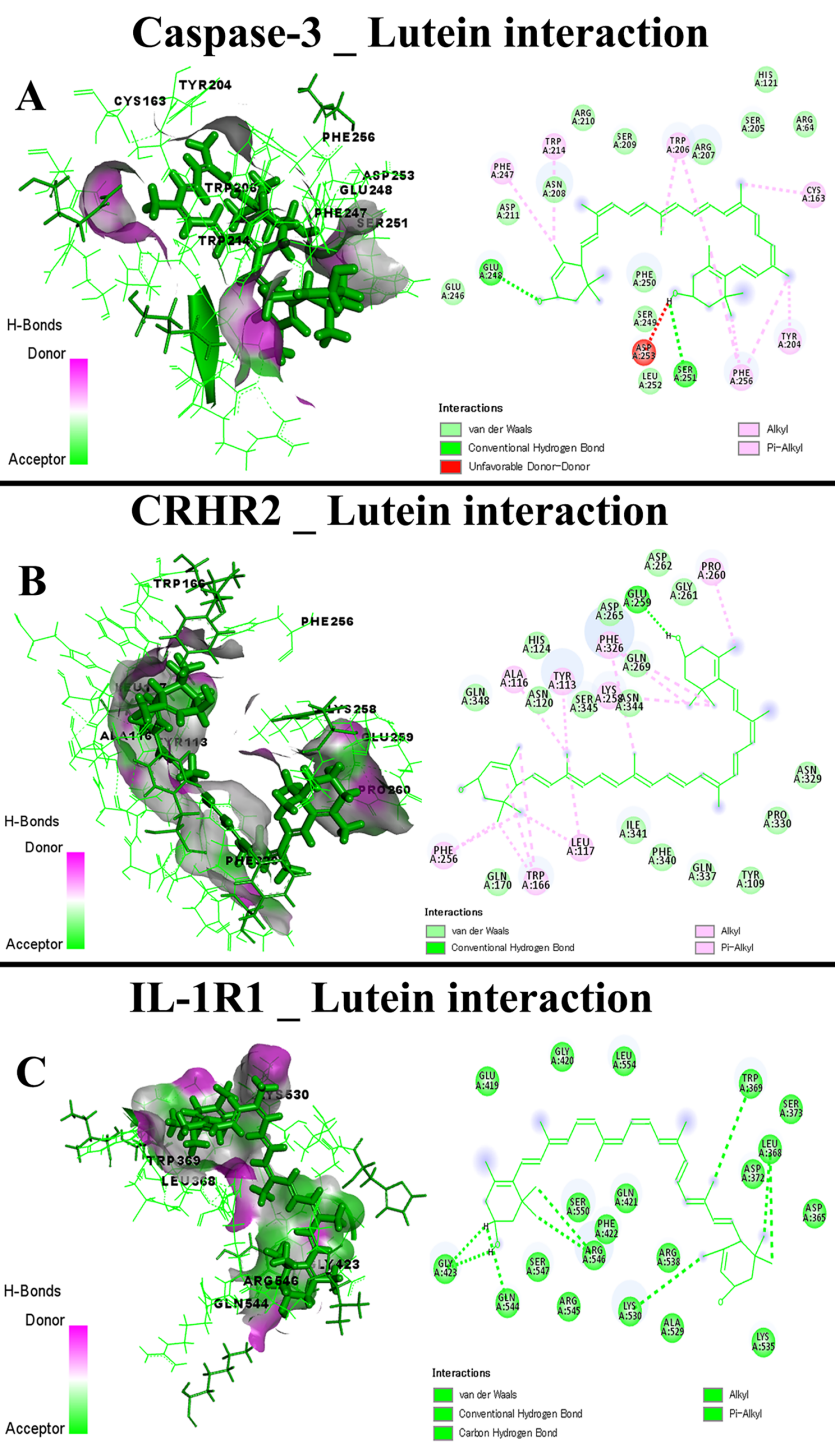
Molecular interaction of chia seed oil active ingredients (Lutein) with caspase-3 (**A**), corticotropin-releasing factor receptor 2 (CRHR2) (**B**), interleukin-1 receptor type 1 (IL-1R1) (**C**).

**Figure 14:**
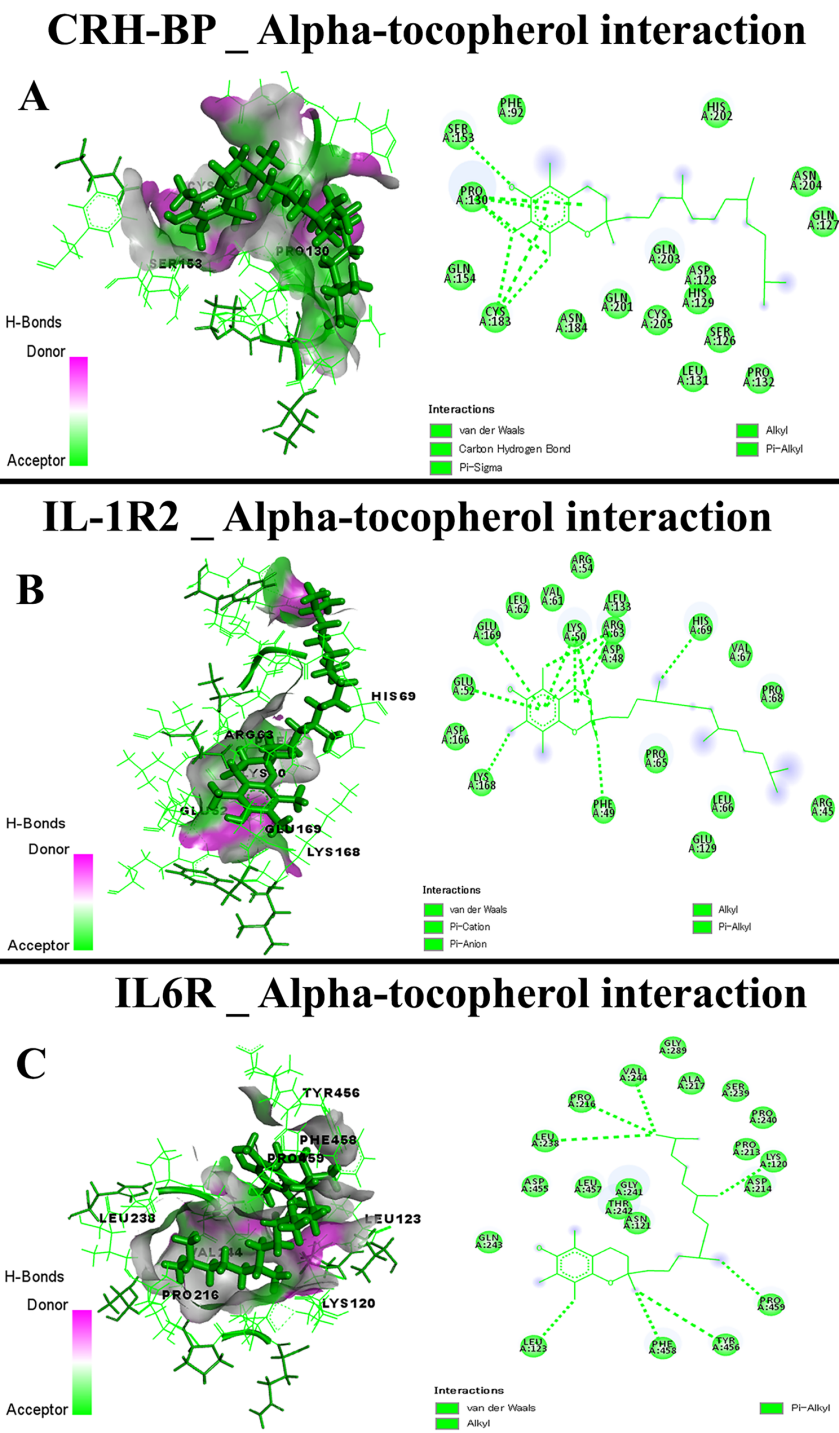
Molecular interaction of chia seed oil active ingredients (Alpha-tocopherol) with corticotropin-releasing hormone binding protein (CRH-BP) (**A**), interleukin-1 receptor type 2 (IL-1R2) (**B**), interleukin 6 receptor, alpha (IL6R) (**C**).

**Figure 15:**
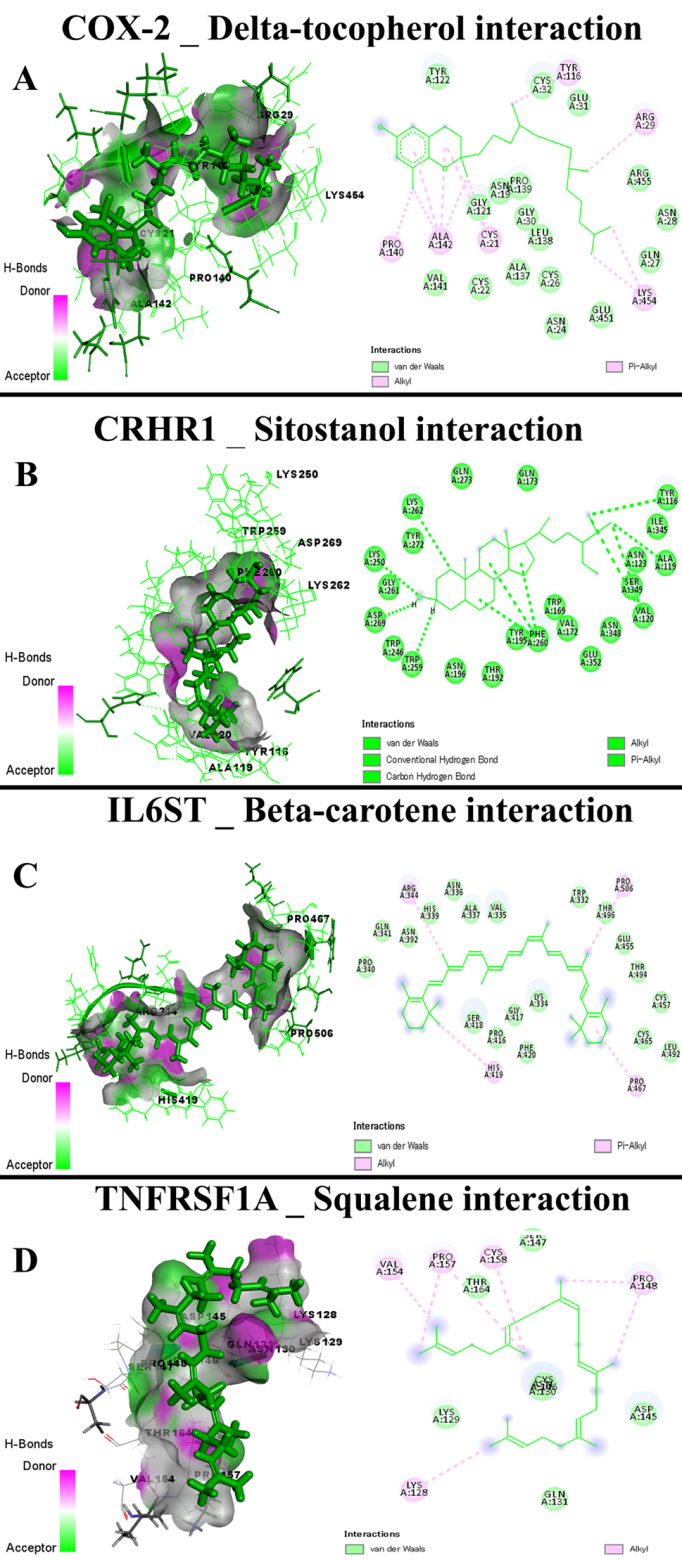
Molecular interaction of chia seed oil active ingredients (Delta-tocopherol) with cyclooxygenase-2 (COX-2) (**A**), (Sitostanol) with corticotropin-releasing factor receptor 1 (CRHR1) (**B**), (Beta-carotene) with interleukin-6 receptor subunit beta (IL6ST) (**C**), and (Squalene) with tumor necrosis factor receptor superfamily member 1A (TNFRSF1A) (**D**).

Data presented in Table 4 showed the docking scores and root-mean-square deviation (RMSD), the average distance among the superimposed protein atoms. The active ingredients of CSO exhibited different scores toward caspase-3, COX-2, CRH-BP, CRHR1, CRHR2, IL-1R1, IL-1R2, IL6R, IL6ST, and TNFRSF1A. The docking interactions of the target proteins with the active ingredients (highest score) of CSO were illustrated in Figs. 12, 13, and 14, showing the bond interaction of the target proteins' amino acids in the binding sites and the active ingredients. Lutein of CSO exhibited a high docking score toward caspase-3, CRHR2, and IL-1R1 (Fig. 13A–C, respectively), while alpha-tocopherol bound well with CRH-BP, IL-1R2, and IL6R (Fig. 14A–C, respectively). Furthermore, delta-tocopherol exhibited a higher affinity to COX-2 (Fig. 15A). In the same manner, sitostanol, beta-carotene, and squalene interacted with CRHR1, IL6ST, and TNFRSF1A binding sites, respectively (Fig. 15B–D, respectively).

**Table 4:** Molecular scores of chia seed oil active ingredients with caspase-3, cyclooxygenase-2 (COX-2), corticotropin-releasing hormone binding protein (CRH-BP), corticotropin-releasing factor receptor 1 (CRHR1), corticotropin-releasing factor receptor 2 (CRHR2), interleukin-1 receptor type 1 (IL-1R1), interleukin-1 receptor type 2 (IL-1R2), interleukin 6 receptor, alpha (IL6R), interleukin-6 receptor subunit beta (IL6ST), and tumor necrosis factor receptor superfamily member 1A (TNFRSF1A).

Molecules	Caspase-3		COX-2		CRH-BP		CRHR1		CRHR2		IL-1R1		IL-1R2		IL6R		IL6ST		TNFRSF1A	
	S	RMSD	S	RMSD	S	RMSD	S	RMSD	S	RMSD	S	RMSD	S	RMSD	S	RMSD	S	RMSD	S	RMSD
Alpha-linolenic acid	−5.95	1.55	−7.98	1.27	−5.34	2.93	−6.38	1.25	−6.38	1.85	−6.34	2.34	−6.92	1.49	−6.82	2.20	−6.29	2.36	−5.22	1.35
Alpha-tocopherol	−6.67	3.56	−8.40	1.62	−6.61	3.11	−7.19	3.07	−7.07	3.06	−6.96	1.20	−7.50	1.46	−7.42	1.67	−7.21	1.87	−5.63	1.61
Arachidonic acid	−6.03	1.15	−7.15	1.22	−5.50	1.77	−6.42	2.49	−6.70	1.67	−6.57	1.97	−6.97	2.43	−6.57	1.93	−6.62	1.67	−5.21	2.54
Caffeic acid	−4.63	1.02	−5.08	1.49	−4.19	1.57	−4.64	0.76	−4.56	1.21	−4.74	1.86	−5.01	2.03	−5.14	1.85	−4.50	2.29	−4.12	3.57
Chlorogenic acid	−5.55	2.00	−7.10	1.52	−5.51	1.17	−6.44	1.21	−5.89	1.44	−6.35	0.87	−7.32	2.22	−6.57	1.60	−5.76	1.10	−4.81	1.93
Delta-tocopherol	−6.18	2.10	−9.03	3.02	−5.69	1.16	−6.80	1.35	−7.17	1.75	−6.94	1.70	−7.16	1.38	−7.66	2.49	−7.12	1.02	−5.61	2.62
Gamma-linolenic acid	−5.72	1.79	−6.92	1.52	−5.23	2.64	−6.49	2.27	−7.09	1.48	−5.71	1.83	−6.91	1.39	−6.59	2.10	−6.41	1.65	−5.73	2.60
Kaempferol	−4.88	4.48	−6.64	1.34	−4.67	2.32	−5.58	1.02	−5.59	1.26	−5.89	2.55	−5.42	2.43	−5.55	1.48	−5.42	1.15	−4.86	2.22
Linoleic acid	−5.98	1.53	−7.46	1.98	−5.35	1.06	−6.41	1.93	−6.69	1.19	−6.31	2.58	−6.27	2.23	−6.28	1.47	−6.48	1.94	−5.21	1.38
Lutein	−6.92	5.18	−7.87	0.97	−6.27	2.27	−5.87	1.98	−8.26	4.14	−7.71	2.52	−5.95	2.47	−7.26	2.16	−6.69	1.84	−4.54	2.43
Myricetin	−5.24	2.26	−7.01	2.00	−4.97	1.13	−5.65	0.96	−5.83	1.63	−5.51	1.19	−5.61	1.06	−5.21	1.27	−5.29	1.27	−4.43	4.73
Oleic acid	−6.31	0.97	−7.33	1.64	−5.47	1.78	−6.65	1.73	−6.66	2.66	−6.50	1.47	−6.36	0.81	−6.44	1.23	−6.59	1.85	−5.25	3.32
Palmitic acid	−5.97	1.34	−7.82	1.38	−5.46	1.22	−6.62	1.12	−6.52	2.36	−6.30	1.60	−6.20	1.55	−6.55	2.30	−6.16	2.12	−6.17	1.00
Palmitoleic acid	−5.90	1.19	−7.40	1.79	−5.06	2.64	−6.42	1.88	−6.22	1.63	−5.90	2.24	−6.24	1.32	−6.53	1.41	−6.28	0.98	−5.79	1.63
Quercetin	−5.04	2.65	−6.68	1.47	−4.69	0.92	−5.39	1.84	−5.35	2.16	−5.38	1.44	−5.29	1.56	−5.55	2.12	−5.21	1.31	−4.38	1.89
Rosmarinic acid	−5.68	1.72	−7.64	2.43	−5.78	2.53	−6.73	2.07	−6.30	2.86	−6.09	3.42	−5.76	1.46	−6.65	2.64	−6.13	1.27	−5.02	2.16
Squalene	−6.54	2.27	−6.46	2.80	−6.23	3.19	−6.72	1.88	−7.64	2.70	−7.57	1.58	−5.92	1.52	−7.32	3.01	−6.56	0.92	−6.52	2.63
Sitostanol	−5.96	2.46	−6.52	2.75	−5.22	1.90	−8.03	1.44	−5.77	1.37	−6.34	2.89	−6.03	1.09	−7.01	3.14	−7.12	1.09	−5.83	1.04
Stigmasterol	−6.42	3.75	−7.44	3.68	−5.48	4.06	−7.08	1.52	−6.33	1.29	−6.14	0.95	−6.08	1.61	−7.35	3.50	−6.89	1.60	−5.14	1.21
Vaccenic acid	−6.12	2.24	−7.18	2.08	−5.54	3.02	−6.83	1.25	−6.76	1.74	−6.10	1.36	−6.46	2.31	−6.69	2.53	−6.49	1.32	−5.26	3.64
Arachidic acid	−6.64	2.33	−7.66	1.28	−5.62	3.11	−7.07	1.71	−7.04	1.60	−7.01	1.44	−6.66	1.64	−6.69	2.02	−6.77	1.51	−5.71	1.52
Gamma-tocopherol	−6.46	1.18	−9.02	1.06	−6.07	1.74	−6.56	1.62	−6.87	1.50	−7.06	1.15	−7.27	1.72	−7.31	2.00	−6.98	2.09	−5.63	2.26
Stearic acid	−6.18	1.16	−7.73	0.78	−5.41	1.43	−6.54	1.45	−6.01	0.88	−6.73	1.06	−7.12	0.99	−6.57	1.54	−6.22	1.60	−5.64	1.99
Beta-sitosterol	−6.38	2.38	−6.93	1.16	−5.40	2.25	−7.32	1.33	−6.03	1.63	−6.07	2.06	−6.43	2.12	−7.32	4.29	−6.93	1.60	−5.86	1.24
Beta-carotene	−6.66	2.75	−3.81	0.80	−4.75	4.46	−7.45	3.47	−7.35	3.83	−5.17	0.43	−6.25	0.81	−6.66	3.14	−7.35	2.80	−3.76	0.49

*S* docking score, *RMSD* root-mean-square deviation.

### Pearson correlation matrix between brain CRF gene expression, serum corticosterone, proinflammatory cytokines, brain oxidative stress markers, and immunoexpression

**Figure 16:**
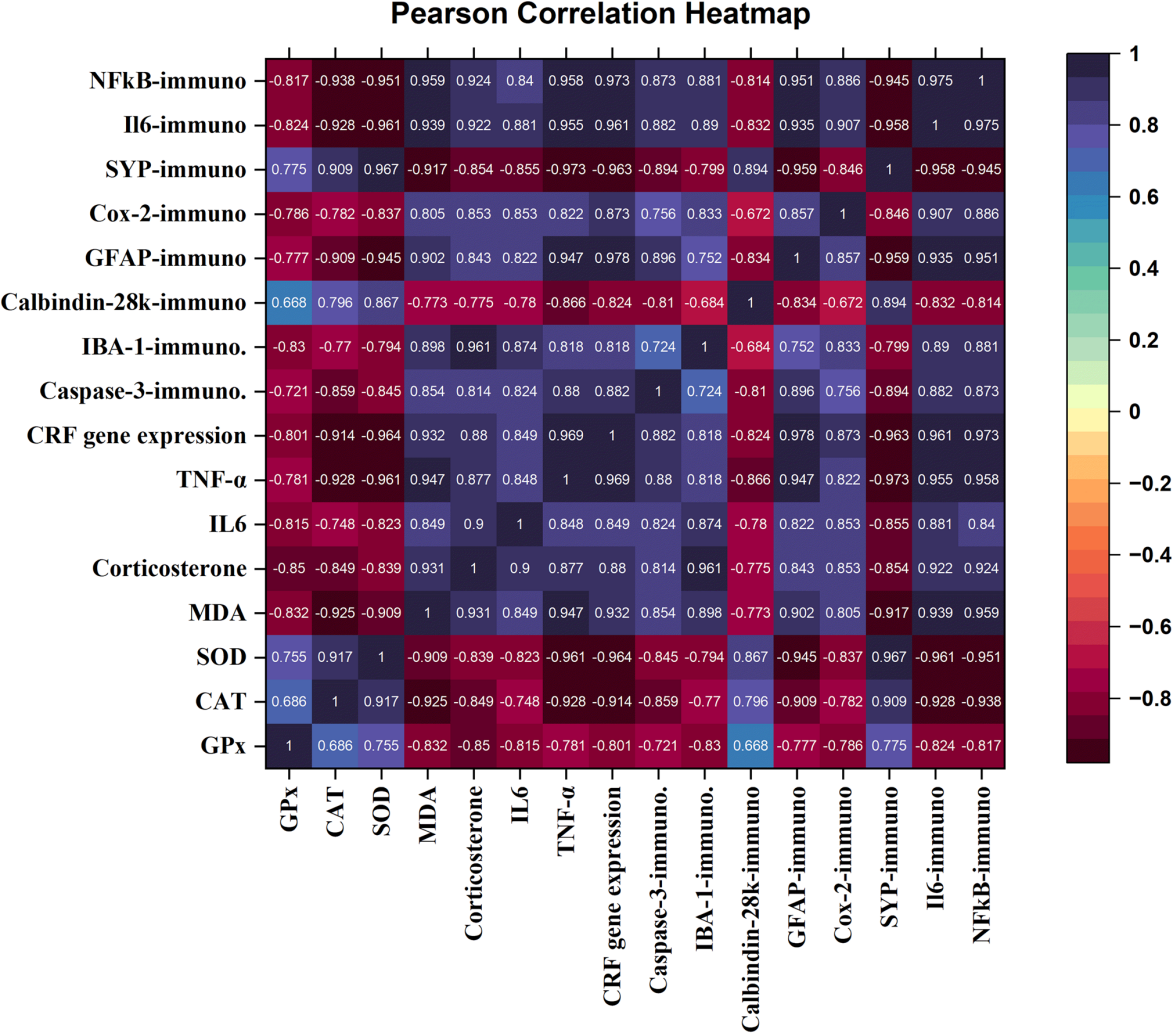
The correlation matrix heatmap demonstrates the Pearson correlation coefficient values between brain CRF gene expression, serum corticosterone, proinflammatory cytokines, brain oxidative stress markers, and immunoexpression. The positive values are in blue, and the negative values are in purple. It ranges from 1 to −1, whereby 1 means a strong positive correlation between the tested variables, −1 indicates a strong negative correlation between the tested variables, and 0 indicates that there is no correlation. *GPx* glutathione peroxidase, *CAT* catalase, *SOD* superoxide dismutase, *MDA* malondialdehyde, *IL-6* interleukin 6, *TNF-α* tumor necrosis factor-alpha, *CRF* corticotrophin-releasing factor, *IBA1* ionized calcium binding adaptor molecule, *GFAP* glial fibrillary acidic protein, *COX-2* cyclooxygenase-2, *SYP* synaptophysin, *NF-κB* nuclear factor kappa light chain enhancer of activated B cells (the reader is directed to the online version of this article for the color references interpretation of in this legend).

As demonstrated in the correlation matrix heat map in Fig. 16, a significant (p < 0.01) positive correlation was found between the CRF gene expression and brain immunoexpression of caspase-3, COX2, IL6, GFAP, IBA-1, and NFκB but a significant (p < 0.01) negative correlation with brain immunoexpression of calbindin-28k and SYP. Furthermore, the CRF gene expression had a significant (p < 0.01) positive correlation with serum corticosterone and proinflammatory cytokines, including IL6 and TNF-α levels. Whereas a significant (p < 0.01) negative correlation was found between the CRF gene expression and the brain antioxidants (SOD, CAT, and GPx), but a significant (p < 0.01) negative correlation with lipid peroxidation indicator (MDA).

## Discussion

**Figure 17:**
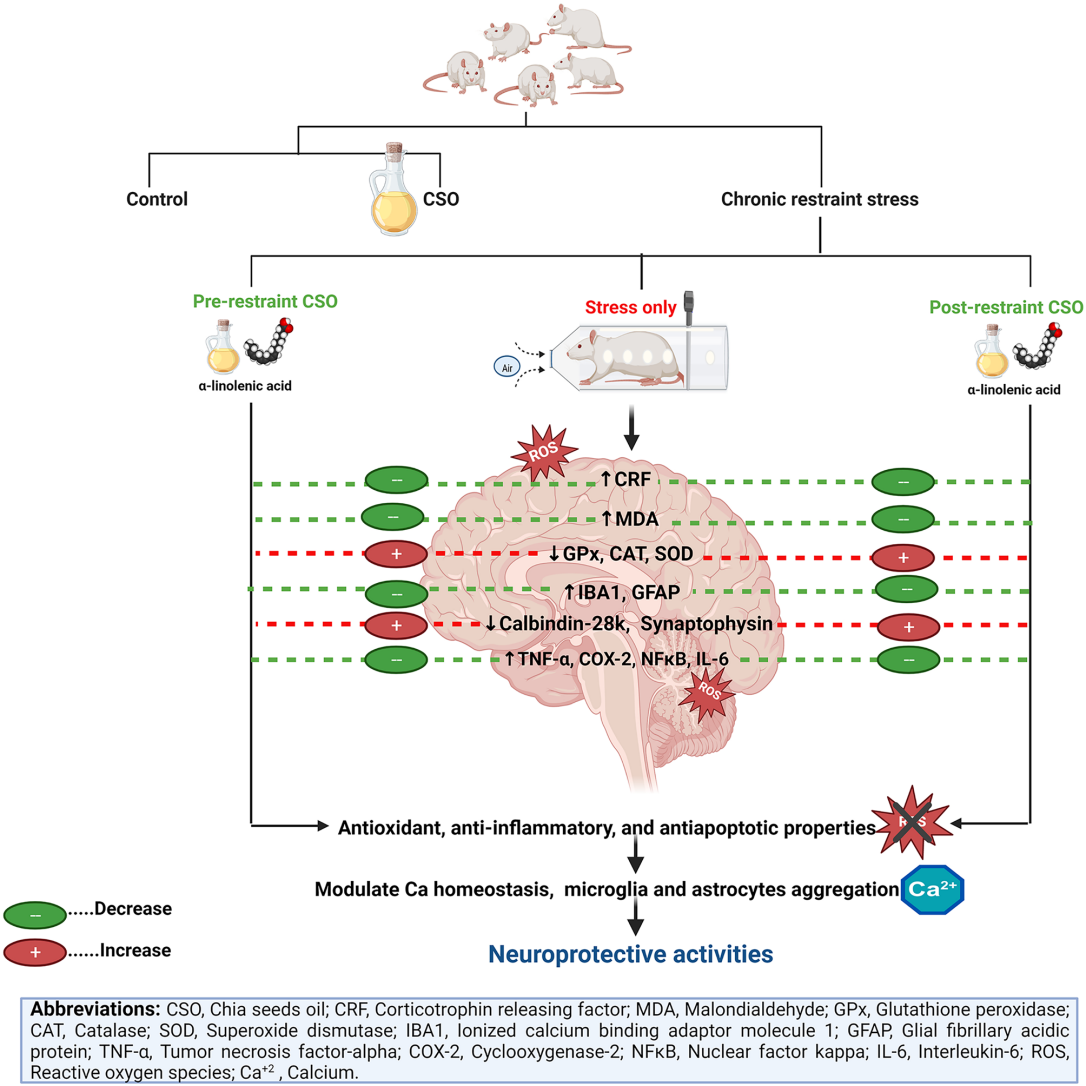
Diagram illustrating the protective influences of chia seed oil (CSO) against chronic immobilization stress and the detected mechanisms.

Our findings reveal that (i) chronic restraint stress exerted severe neuro-disturbance in rats' brains through the stimulation of apoptotic, inflammatory, and oxidative signaling pathways; (ii) chronic immobilization induced disturbance in calcium homeostasis, stimulation of astrocytes and microglia infiltration, as well as diminishing of synaptophysin release, could share part of brain injury mechanisms; (iii) CSO administration for stressed rats improved the antioxidative status and reduced oxidative marker; (iv) CSO exhibit a marked lowering in the immuno-expression of caspase-3, ionized calcium binding adaptor molecule, GFAP, NFkB, IL-6, and COX-2, and a significant elevation in calbindin-28k and SYP expression. The promising neuroprotective activities of CSO are presented in Fig. 17.

Stress is a common cause of many disorders and diseases^60^. In mammals, the stress pathophysiology induces several dysfunctions strongly correlated to alterations in the immune status, hormone levels, and cytokines profile^61^. Stress initiates the HPA axis activation and stimulates stress-related hormone secretion, such as GCs, which are secreted from the adrenal cortex to the bloodstream and reach the brain via the blood–brain barrier^62^.

In the current study, the corticosterone concentrations in the restraint group are significantly augmented relative to the control group, which may be due to response stimulation of its secretion from the adrenal cortex through HPA and SAS neuroendocrine. A similar result was recently reported by numerous reports^63–66^, where those authors concluded that chronic immobilization significantly increases the corticosterone levels in the serum of the exposed group. On the contrary, stressed groups pre or post-treated with CSO exhibited a significant reduction in serum corticosterone concentrations related to the restraint group. This finding indicates the ameliorative role of CSO in the modification of the stressful status of the subjected rats. This ameliorative impact might be due to the presence of essential fatty acids such as ALA, an essential omega-3 PUFA derived from plants, which has anti-inflammatory and oxidative stress modulators. These findings agreed with the result of Schreyer, et al.^67^, who stated that the administration of chia seeds in mice attenuated corticosterone levels in diet-induced metabolic stress and Alzheimer's disease. It also documented that docosahexaenoic acid (DHA)-enriched fish oil, an omega-3 fatty acid, suppressed the increased serum corticosterone concentration in chronically stressed rats^68^.

The increased stress hormones could modulate the release of cytokine chemicals secreted by T cells (T helper cells: a type of T lymphocytes)^69^. Cytokines are chemically defined as glycosylated polypeptides, which determine and regulate the immune response nature^70^. In the present findings, the pro-inflammatory mediators TNF-α and IL-6 are significantly raised in the immobilized stressed rats, indicating tissue damage and inflammatory response that could be induced by oxidative stress; this result agrees with the result of Miller, et al.^71^, who informed that chronic immobilization induces a marked elevation of TNF-α and IL-6 levels in sera as well as the result of Omer, et al.^72^ who concluded that chronically immobilized rats exhibit a significant elevation in the pro-inflammatory cytokine TNF-ɑ, IL-1ß, and IL-6. The administration of CSO before and after stress ameliorates the negative impact of chronic immobilization by lowering the IL-6 and TNF-α levels, which were reduced markedly in the CSO pre-restrained group. This ameliorative effect could be due to the antioxidant activity of ALA, which improves oxidative stress-induced damage and modulates the immune response. A comparable result was achieved by Mohamed, et al.^25^, who noted that CSO and chia seeds mucilage diminished oxidative stress, reduced TNF-α, and exhibited anti-inflammatory effects. It also recognized that CSO has anti-inflammatory and antioxidant effects by lowering inflammatory chemical mediators such as TNF-α and IL-6 as well as ROS (NO and H_2_O_2_) levels^73,74^.

Plant products are an excellent source of natural antioxidants because of their highly effective bioactive compounds, lower cost, and satisfactory safety^75^. In the existing trial, the levels of antioxidant enzymes in neural tissues of the restraint group, including GPx, CAT, and SOD, showed a significant reduction, together with a significant upsurge in the MDA levels related to their values in the control and CSO groups. These results are mostly related to the immobilization-induced oxidative stress, which might result in ROS over-accumulation that initiates lipid peroxidation and tissue damage. Several studies also reported that the activity of the antioxidant biomarkers, for instance, SOD, GPx, GSH, and CAT, were significantly reduced in hepatic and brain tissues in the rat group exposed to immobilization stress^76–78^. In contrast, the MDA level, an oxidative status biomarker, was significantly elevated^72,79^. In the current investigation, there was a marked elevation in the SOD, GPx, and CAT levels and a significant decline in MDA level in brain homogenate of CSO pre and post-restrained rats, indicating the modulator effect of CSO treatment to enhance the balance between the production of antioxidants and oxidants to vanish the oxidative damage and promote homeostasis and this effect may be due to their high content of ALA or omega-3. Similarly, Himanshu, et al.^80^ observed that the supplementation of flax seeds oil, a herbal source of omega 3 in rams, increases antioxidant activity and reduces the induced oxidative stress. As well, walnut oil contains high levels of essential PUFAs, including ALA and omega-3, which protect from nitrite-induced oxidative stress by a significant decrease of MDA and elevation of antioxidant activities (SOD, GSH, and CAT)^81^.

During the stress response initiation, the system of CRF has a crucial effect on the pathophysiological mechanism of depression and anxiety and in modulating the body's reaction to external stimuli^82^. The results attained from the existing experiment displayed that the brain CRF expression was markedly raised in the stressed group; this increase may be due to CRF playing an essential role in the organizing of the HPA axis during the stress response, the same result reported by Varodayan, et al.^83^ who found that stress induces neuroimmune responses that elevate stress peptide (CRF) following chronic immobilization stress. While in the CSO- pre and post-restraint rats, there was a marked reduction in CRF gene expression in rats' brains relative to the restraint group, which may be due to the enrichment of CSO with PUFAs, mainly ALA, a potent antioxidant and antioxidative stress. This result agrees with the results of Jisu^84^, who said that omega-3 PUFA decreases CRF expression and exhibits antidepressant activity through the HPA axis modification. It is also documented that oral treatment of ALA in mice can reduce oxidative stress and inhibit neural inflammatory reactivity in the brain cortex^85^.

In the existing experimentation, chronic immobilization stress provoked several histopathological lesions in the different brain compartments. The most prominent lesions were in the form of neuronal death and glial cell reactions. These lesions could be primarily related to restraint-induced oxidative and inflammatory injuries, as mentioned above, which in turn induced alteration and dysfunction of the cytoskeleton and mitochondria^86^. The reported neuronal necrosis might be explained by the ability of the regenerated ROS to impair vascular function^87^. In addition, the current investigation showed the cornus ammonis 4 (CA4) pyramidal cell degeneration and disorganization, as well as dentate gyrus neuronal apoptosis. Sun, et al.^88^ proposed that memory impairment may be accompanied by the death of pyramidal cells. Along the same line, Woo, et al.^89^ concluded that chronic restraint stress is a crucial psychological element that may impair memory and learning in the hippocampus. Hence, the attenuation of these reported lesions, particularly in prior treatment with CSO, might be attributed to its antioxidant as well as anti-inflammatory abilities^73,74^.

In numerous trials, authors informed that chronic immobilization stress could modify the immune status via the cell death receptor Fas-induced apoptosis and Toll-like receptors (TLRs)^90,91^. Furthermore, chronic immobilization can result in the overproduction of free radicals and mitochondrial dysfunction, resulting in the consequent production of apoptotic proteins such as caspase 3. In the current study, marked positive immunoexpression of caspase-3 was reported in the restraint group, which agrees with the detections of Kwatra, et al.^92^, who suggested that chronic restraint stress activated the apoptotic signal resulting in neurobehavioral changes in the mice. The anti-inflammatory and antioxidant activities of CSO could clarify its antiapoptotic impact, as exhibited by the mild caspase-3 immunoexpression in immobilized rats pre or post-restraint with CSO. Similarly, it concluded the beneficial antiapoptotic impact of the aqueous extract of chia seeds^93^.

The findings of the existing study revealed that the immunohistochemical expression of pro-inflammatory markers, including NF-kB, COX-2, and IL-6- was increased in the restraint group relative to the control group. Besides, the molecular docking assessment evidenced the anti-inflammatory effect of CSO active ingredients (lutein, alpha-tocopherol, delta-tocopherol, sitostanol, beta-carotene, and squalene) through binding with the apoptotic enzymes and inflammatory cytokines' receptors (caspase-3, CRHR2, and IL-1R1, CRH-BP, IL-1R2, IL6R, COX-2, CRHR1, IL6ST, and TNFRSF1A) binding sites. The most probable explanation for these findings is that chronic immobilization stress leads to increased ROS regeneration with subsequent neuronal injuries and brain inflammation^86^. As previously concluded, injuries induced by oxidative stress may result in increased COX-2 expression^94^. In addition, Salehpour, et al.^95^ concluded a significant increase in NF-kB in chronic restraint stress. On the contrary, Miller, et al.^71^ concluded that chronic stress causes a significant reduction in IL-6, this difference could be attributed to the variation in both the cause and the interval of the stress. In the present trial, prior and post-treatment of stressed rats with CSO showed a significant reduction of these pro-inflammatory cytokines. Similar findings were described by Martínez and Segura^74^, who evaluated the neuroprotective effect of bioactive peptides obtained from chia seeds on the HMC3 microglial cells' pro-inflammatory modulation.

Calbindin-28k is a calcium-binding protein localized intracellularly that may promote transcellular calcium flow. In the brain, it was suggested that calbindin-28k is crucial for inhibiting neuronal cell death and sustaining intracellular calcium homeostasis^96^. Although the exact function of calbindin-28k seems indistinct, its function as a buffered protein and a carrier enables Ca^2+^ transcellular transportation and keeps its concentration in the optimum range throughout the transportation proposed^97^. In this investigation, the calbindin-28k immunohistochemical expression was reduced in the restraint group. Conversely, a marked elevation in the area % of calbindin-28k immunoreactivity was observed in rats subjected to CSO. This finding may propose that chronic stress might initiate changes in Ca^+2^ handling. This finding is in line with the findings of Leem and Chang^98^, who noticed that chronic stress led to reducing immunohistochemical calbindin-28k expression in hippocampal cornus ammonis 1 (CA1) cells. This finding suggests the relationship between chronic stress and disrupted intracellular calcium homeostasis; many studies have shown that synaptic plasticity in chronic stress-induced neural injury is greatly influenced by calcium channel-dependent depolarization and kinases, including CaMKs^99^. On the contrary, rats treated with CSO before or after chronic stress showed elevation in Calbindin-28k levels; this elevation could be attributed to the beneficial content of ALA or omega-3 in the chia seeds. Further studies are needed to discuss the possible pathways by which CSO can influence calbindin-28k in brain tissues.

IBA1 is a calcium-binding protein molecule that is specifically expressed in macrophage and microglia cells. It contributes to membrane ruffling, actin bundling, and phagocytosis in activated microglial cells. In the present work, a marked elevation in the immuno-expression of IBA1 in the restraint group was evident; this increase could be attributed to the activation of microglia in the hippocampal area under chronic restraint stress. A similar finding was detected before by Tynan, et al.^100^ and Bian, et al.^101^, who informed that the stressed animals revealed a significantly elevated IBA1-reacted cells number in the cornus ammonis 3 (CA3) and prelimbic areas of the hippocampus. Conversely, the CSO group showed a marked decline in IBA1 immuno-expression. A similar finding was obtained recently by Syeda, et al.^31^; these authors attributed this finding to the presence of alpha-linolenic acid, which is a precursor to the eicosapentaenoic acid (EPA), DHA, and long-chain fatty acids. Omega-3 PUFAs have neuroprotective effects and have the ability to alleviate several neurological and neurodegenerative alterations^102^.

SYP, a Ca^2+^-binding synaptic vesicle membrane protein^103^, acts as a common indicator protein of nerve endings, particularly the pre-synaptic one^104^. SYP is necessary for neurotransmitter liberation and vesicle fusion^105^. In several animal trials, stress has been confirmed to prompt the SYP mRNA expression down-regulation^106,107^. In a similar context, our findings demonstrated a significant decline in the immunoexpression of SYP in brain tissues in the restraint group. This finding agreed with that of Xu, et al.^108^, who concluded that repeated immobilization stress reduces the SYP expression in the hippocampus. On the contrary, pre or post-restraint with CSO, particularly in the CSO pre-restraint rats, significantly accelerates the recovery processes with SYP. No available data are present to discuss such beneficial effects. However, the presence of omega-3 PUFAs in CSO could be shared in the required explanation due to its neuroprotective properties and its possible effect in treating several neurodegenerative alterations^102^.

GFAP, a specific astrocyte marker, facilitates the preservation of the cytoskeletal architecture and glial cells' mechanical potential and supports adjacent neurons^109^. It was suggested that GFAP has an astrocyte-like function^110^. A prior investigation by Roberts, et al.^111^ indicated that higher levels of GFAP refer to astrogliosis because of neuronal and glial damage. It was reported previously in non-myelinating Schwann cells as an indicator of neuroinflammation and brain injury^112^. Furthermore, a marked negative correlation was detected between the CRF gene expression and the brain antioxidant enzymatic system and the immunoreactivity of calbindin-28k and SYP. As reported by Pisoschi, et al.^113^, who illustrated chronic oxidative stress accompanied by oxidative alterations via lipid peroxidation induction and endogenous antioxidant depletion inducing the oxidative stress-related pathology. In contrast, A strong positive correlation was found between the expression of the CRF gene and brain immunoexpression of caspase-3, COX2, IL6, GFAP, IBA-1, NFκB, serum corticosterone, proinflammatory cytokines, and brain lipid peroxidation marker. It is also documented that the chronic stress elevated serum corticosterone concentrations and IBA-1, cleaved caspase-1, NFκB, IL-1β immunoexpression^114^.

In the present experiment, the astrogliosis induced by restraint and, in turn, increased immunoexpression of GFAP was diminished by the administration of CSO. This reduction in immunoexpression could be ascribed to the CSO's antioxidative and anti-inflammatory activities. However, up to now, there are no available studies regarding the beneficial impact of CSO against GFAP. Further studies are required to fill this knowledge gap.

## Conclusion and future perspective

Based on the present outcomes, it could be concluded that the administration of CSO to stressed rats resulted in the amelioration of neurodisturbance in rats' brains through the suppression of inflammatory, oxidative, and apoptotic signaling pathways. In addition, CSO suppressed astrocyte and microglia infiltration, enhanced synaptophysin release, and regulated calcium homeostasis in the brains of stressed rats. To the reader's knowledge, few studies are available concerning the protective efficacy of CSO against neurodisturbance. However, to date, there is no available data about the potential ameliorative impacts of CSO against restraint stress and mRNA gene expression of corticotrophin-releasing factor. The promising neuroprotective activities of CSO have been suggested owing to the anti-inflammatory properties, memory enhancer, and antioxidant promotor effect of α-linolenic acid contained in CSO in high amounts. Future studies are necessary to confirm our present findings, confirm memory impairment and cognitive dysfunction via neurobehavioral tests, and evaluate the therapeutic impacts of CSO in human patients with neurodisturbance.

## Data Availability

All generated or analyzed data throughout this investigation are included in this article.
